# Beyond Incidence and Mortality: Socioeconomic Mediation of Gastric Cancer Disparities in the United States, 1990–2021

**DOI:** 10.5334/aogh.5015

**Published:** 2026-02-04

**Authors:** Yun Seo Kim, Sarah Soyeon Oh, Sung Hwi Hong, Minseo Kim, Dong Keon Yon, Jae Il Shin, Chul S. Hyun

**Affiliations:** 1 Yonsei University College of Medicine, Seoul, Republic of Korea; 2 Institute for Global Engagement & Empowerment, Yonsei University, Seoul, Republic of Korea; 3 Severance Underwood Meta-Research Center, Institute of Convergence Science, Yonsei University, Seoul, Republic of Korea; 4 Cardiovascular Disease Initiative, Broad Institute of MIT and Harvard, Cambridge, MA, USA; 5 Center for Digital Health, Medical Science Research Institute, Kyung Hee University College of Medicine, Seoul, Republic of Korea; 6 Department of Pediatrics, Kyung Hee University College of Medicine, Seoul, Republic of Korea; 7 Section of Digestive Diseases, Department of Medicine, Yale School of Medicine, New Haven, CT, United States; 8 Department of Paediatrics, Yonsei University College of Medicine, Seoul, Republic of Korea

**Keywords:** gastric cancer, disability-adjusted life years (DALYs), United States, state-level disparities, racial/ethnic inequities, socioeconomic mediation, *Helicobacter pylori*

## Abstract

*Background:* Gastric cancer (GC) remains a major global health burden, yet US trends often obscure disparities hidden within national averages. Although incidence and mortality have declined overall, profound geographic, racial, and socioeconomic differences persist. Few studies have systematically examined how demographic composition and social determinants jointly shape GC burden across states.

*Methods:* We analyzed Global Burden of Disease 2021 estimates for GC incidence, mortality, and disability-adjusted life years (DALYs) from 1990 to 2021 across 50 US states and the District of Columbia. Outcomes were stratified by age, sex, race/ethnicity, and sociodemographic index. Multivariable and mediation models assessed how income and education modified racial and ethnic disparities.

*Results:* While national GC rates declined over three decades, the burden remained concentrated in states with large immigrant and low-income populations, including Hawaii, the District of Columbia, Mississippi, and New Mexico. States with higher Asian populations exhibited roughly fourfold greater incidence than those with larger Hispanic populations. Income and education together mediated 22%–31% of racial and ethnic disparities, demonstrating that socioeconomic position—not race alone—drives much of the observed heterogeneity.

*Conclusions:* This state-level sociodemographic analysis reveals the structural underpinnings of US GC inequities within a broader global context of uneven early-life risk and population diversity. By linking racial composition, income, and education to disease burden, it identifies modifiable pathways for prevention and policy action. Viewed as a case study for migrant-receiving countries, these findings underscore the importance of equity-informed strategies—such as *Helicobacter pylori* screening, nutrition interventions, and targeted resource allocation—to address persistent GC disparities globally.

## Introduction

Gastric cancer (GC) remains a major global health problem, ranking fifth in incidence and fourth in cancer-related mortality worldwide, with more than 1,100,000 new cases each year and a disproportionate burden concentrated in East Asia, Eastern Europe, and parts of Latin America [1]. In the United States (US), overall incidence and mortality have declined steadily over the past three decades, with incidence rates falling from 9.6 per 100,000 in 2000 to 5.7 per 100,000 in 2021 [2]. Mortality has decreased across all racial and ethnic groups, with the most rapid declines observed among Asian and Black populations and more modest reductions among American Indian/Alaska Native and Latino populations. These improvements reflect advances in diagnostics, early detection, and eradication of *Helicobacter pylori* (*H. pylori*), a major etiologic factor for GC [3].

Despite these national declines, global GC risk remains highly uneven, and elevated risk persists among populations with early-life exposure in high-incidence regions—a risk that increasingly manifests in migrant-receiving countries such as the United States. Elevated risks among Asian, Hispanic, and Black Americans, along with higher burdens in selected states, are well documented in registry-based epidemiology. However, national-level statistics obscure the depth of inequities that persist beneath aggregate declines in incidence and mortality. Among individuals aged 50 years and older, the incidence of non-cardia gastric cancer (NCGC) among Korean Americans is approximately 49 per 100,000, more than 13 times higher than that observed among the non-Hispanic White population (3.7 per 100,000) [4]. Similarly elevated rates are observed among Vietnamese (6.5-fold higher), Japanese and Chinese (5-fold higher), and non-Hispanic Black (3-fold higher) Americans, underscoring substantial racial and ethnic disparities in disease burden [4]. These patterns closely mirror global incidence gradients and are consistent with the persistence of exposure-linked risk following migration rather than de novo risk acquired in the host country. Although mortality has declined in most groups, the majority of US counties continue to report significantly higher mortality among minoritized populations compared with White populations—99.9% of counties for Black populations, 99.4% for Asian populations, and 88.2% for Latino populations [5].

GC comprises two main anatomical subtypes—cardia (CGC) and non-cardia (NCGC)—with distinct etiologic profiles [1]. While both share risk factors such as smoking and alcohol use, NCGC is strongly associated with *H. pylori* infection and high-sodium diets, exposures that remain prevalent in many high-incidence regions globally and are disproportionately concentrated among immigrant and minoritized populations in the US. Social determinants further exacerbate disparities; for example, predominantly Black neighborhoods have significantly fewer supermarkets than White neighborhoods, with a store-to-population ratio of just 0.5, limiting access to nutritious foods [6]. Traditional Asian diets rich in pickled and salted foods may also contribute to elevated GC risk [7]. Together, these biological, environmental, and structural factors illustrate how global exposure patterns intersect with local social context to shape observed disparities in the US.

While interest in GC disparities has increased in recent years, most US studies have focused on aggregated racial and ethnic categories, often overlooking heterogeneity within groups as well as the roles of migration history and socioeconomic position [5]. Similar limitations characterize much of the global literature, where reliance on national averages can obscure within-country inequities by region, ethnicity, or socioeconomic status. Geographic analyses have emphasized mortality but have rarely integrated incidence, disability-adjusted life years (DALYs), or social determinants—metrics that together provide a more comprehensive assessment of cancer burden [8]. Recent Global Burden of Disease (GBD)-based analyses have described temporal and spatial patterns in US GC burden from 1990 to 2021 [8, 9], paralleling international GBD efforts that document persistent global inequalities in GC burden across countries and regions [10]. However, these studies have remained largely descriptive and have not examined how demographic or socioeconomic factors mediate observed disparities.

The United States offers a distinctive analytic context for global health research as a migrant-receiving country in which exposure-linked risks acquired abroad intersect with heterogeneous health systems, policies, and social environments. Our recent multi-state analysis demonstrated that GC burden is concentrated in seven US states with large immigrant populations, underscoring the regional and ethnic heterogeneity concealed by national averages [11]. Building on this work, the present study provides the first comprehensive state-level assessment of US GC burden from 1990 to 2021 using GBD 2021 data. Because GBD methodology is standardized and applied globally, this analytic framework is directly transferable to other countries and regions with available subnational data. By examining incidence, mortality, and DALYs across all 50 states and the District of Columbia and linking outcomes to racial/ethnic composition, income, and education, we move beyond descriptive epidemiology to interrogate the structural and social drivers of inequities.

This approach is important for three reasons. First, DALYs capture the total impact of GC by integrating premature mortality and years lived with disability, providing a more complete measure of burden than incidence or mortality alone. Second, state-level disaggregation identifies where disparities are most concentrated, enabling policymakers to target the highest yield opportunities for prevention. Third, by examining how income and education mediate racial and ethnic disparities, this study highlights actionable levers for intervention that extend beyond biology or demographics. Taken together, these findings position the US experience as a case study of how global exposure patterns, migration, and socioeconomic context interact to shape cancer inequities, offering insights relevant to GC prevention efforts worldwide. In doing so, we provide an updated and integrated view of US GC burden and a framework for targeted, equity-informed prevention strategies, including *H. pylori* screening and eradication and nutrition-focused interventions in communities where the burden remains highest.

## Methods

### Data sources and case definition

We used de-identified estimates from the GBD 2021 study, which are publicly available through the Global Health Data Exchange (http://ghdx.healthdata.org/gbd-2021). GC outcomes included incidence, deaths, prevalence, years of life lost (YLLs), years lived with disability (YLDs), and DALYs for all 50 US states and the District of Columbia from 1990 to 2021. Results were reported by sex and five-year age groups from 15–19 years through ≥95 years. GC was defined using ICD-10 codes C16–C16.9, D00.2, and D13.1, and ICD-9 codes 151–151.9, 211.1, and 230.2. This study adhered to the Guidelines for Accurate and Transparent Health Estimates Reporting (GATHER; eTable 1). Because all data were anonymized and publicly available, institutional review board approval was not required, and the analysis was conducted in accordance with the Declaration of Helsinki.

### Estimation of fatal and non-fatal outcomes

Fatal and non-fatal estimates were generated using standard GBD methodology. Data inputs included vital registration, verbal autopsy, and cancer registry sources. Mortality-to-incidence ratios were estimated with spatiotemporal Gaussian process regression, and mortality was modeled using the Cause of Death Ensemble (CODEm) framework. YLLs were calculated by multiplying deaths by standard life expectancy at each age. Incidence was derived from mortality estimates, and prevalence was estimated by applying survival probabilities from the Surveillance, Epidemiology, and End Results (SEER) program. Prevalence was partitioned into diagnosis and primary therapy, controlled, metastatic, and terminal phases, and YLDs were calculated by multiplying phase-specific prevalence by disability weights. DALYs were obtained by summing YLLs and YLDs. Uncertainty was propagated through all steps, and 95% uncertainty intervals (UIs) were defined as the 2.5th and 97.5th percentiles of 500 draws.

### Sociodemographic variables

Sociodemographic Index (SDI) values were obtained from GBD 2021, and state-level variables—including racial/ethnic composition, aggregate household income (inflation-adjusted to 2021 US dollars), and educational attainment (proportion of adults ≥25 years with a bachelor’s degree or higher)—were drawn from the 2021 American Community Survey (US Census Bureau). Race and ethnicity were harmonized into four mutually exclusive categories: Hispanic, non-Hispanic White, non-Hispanic Black, and non-Hispanic Asian. Smaller groups were excluded due to limited population size.

### Statistical analysis

Associations between burden estimates and SDI were examined using Spearman’s rank correlation. Multivariable linear regression was used to assess relationships between state-level racial/ethnic composition and GC burden, and extended models incorporated income and education. Mediation analyses were conducted to test whether income and education partially explained these associations, using nonparametric bootstrapping with 1000 replicates to generate 95% confidence intervals. Because GBD estimates are not stratified by race or ethnicity, findings represent ecological correlations at the state level and should not be interpreted as individual-level risks. All analyses were performed in R (version 4.5.1, R Foundation for Statistical Computing). Commonly used packages included dplyr, tidyr, ggplot2, lavaan, and usmap. Exact packages and the R session information are provided in the Supplementary Materials. Statistical significance was defined as *p* < 0.05, or when 95% UIs of percentage change estimates excluded zero.

### Data availability statement

All data used in this study are publicly available. GC estimates were obtained from the GBD 2021 database (http://ghdx.healthdata.org/gbd-2021), and sociodemographic variables were extracted from the US Census Bureau’s American Community Survey (https://data.census.gov). Additional raw or processed data generated during the analysis are available from the corresponding author upon reasonable request.

## Results

### Burden of GC in the US

In 2021, there were 28,458.22 [95% UI: 26,390.25–29,870.53] incident cases, 16,444.97 [15,029.59–17,351.08] deaths, and 363,139.06 [342,100.10–377,924.87] DALYs attributed to GC in the United States (Table 1). The age-standardized incidence rate declined by 36.97% [38.82–35.11] from 1990 to 2021, reaching 5.07 [4.74–5.30] per 100,000 in 2021 (eFigure 1). Similarly, the mortality rate decreased to 2.84 [2.62–2.98] and the DALY rate to 69.16 [65.84–71.77], representing reductions of –47.04% [–48.71 to –45.33] and –44.63% [–46.23 to –43.02], respectively.

**Table 1:** State-level incidence, deaths and DALYs of gastric cancer (1990–2021).

	**Incidence (95% UI)**			**Deaths (95% UI)**			**DALYs (95% UI)**		
	**1990**	**2021**	**Percentage change, 1990–2021**	**1990**	**2021**	**Percentage change, 1990–2021**	**1990**	**2021**	**Percentage change, 1990–2021**
**United States of America**									
**Absolute number, thousands**	25.71 [24.11–26.59]	28.46 [26.39–29.87]	10.69% [7.15% to 14.23%]	17.32 [16.09–17.98]	16.44 [15.03–17.35]	−5.22% [−8.10% to −2.28%]	383.16 [366.57–393.93]	363.14 [342.10–377.92]	−5.22% [−8.10% to −2.28%]
**Age-standardized rate, per 100,000**	8.04 [7.57–8.30]	5.07 [4.74–5.30]	−36.97% [−38.82% to −35.11%]	5.36 [4.99–5.55]	2.84 [2.62–2.98]	−44.63% [−46.23% to −43.02%]	124.90 [120.01–128.28]	69.16 [65.84–71.77]	−44.63% [−46.23% to −43.02%]
	**Incidence (95% UI)**			**Deaths (95% UI)**			**DALYs (95% UI)**		
**Top five states, age-standardized rate, 2021**									
	Hawaii		7.88	Hawaii		4.31	Hawaii		106.00
	Louisiana		6.70	Mississippi		3.92	Mississippi		98.88
	Mississippi		6.05	Louisiana		3.69	Louisiana		92.79
	New Mexico		6.02	District of Columbia		3.66	New Mexico		92.65
	Alaska		6.01	Alabama		3.57	District of Columbia		91.58
**Bottom five states, age-standardized rate, 2021**									
	Idaho		3.82	Vermont		2.16	Idaho		49.93
	Vermont		3.96	Iowa		2.17	Vermont		51.09
	Utah		4.03	Idaho		2.19	Iowa		51.52
	Iowa		4.10	Oregon		2.20	Utah		51.56
	Oregon		4.17	Washington		2.24	Washington		52.68

DALYs: disability-adjusted life years.

See Table 1 for a summary; full state-level results with 95% UIs are provided in Supplementary Table S1.

**Figure 1:**
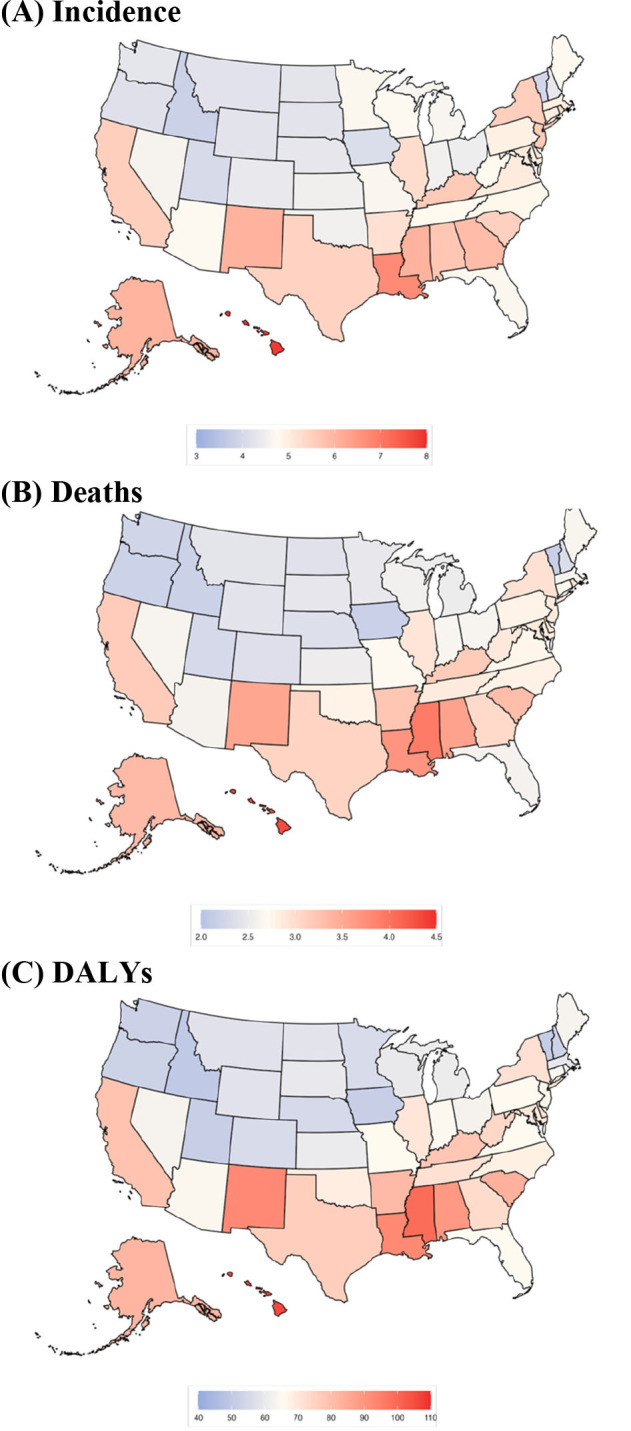
Age-standardized **(A)** incidence, **(B)** death, and **(C)** DALY rate of GC in the United States, 2021. DALYs = Disability-adjusted life years.

All 50 states and DC showed declines over the study period (eTable 2). However, regional disparities were evident (Figure 1, eFigure 2). Northern states such as Vermont, Idaho, and Iowa had the lowest DALY rates in 2021 (49.93 [42.12–58.06], 51.09 [44.65–59.36], and 51.52 [42.26–61.36], respectively), while southern states such as Mississippi and New Mexico had nearly double the burden (92.65 [77.82–108.62] and 98.88 [83.42–117.69]; Table 1). These higher burdens reflected smaller declines in DALY rates from 1990 to 2021 (Mississippi: −30.72% [−42.12 to −16.69]; New Mexico: −32.49% [−43.68 to −20.36]) compared to the northeast, where seven of nine states experienced declines of more than 50%. Hawaii and DC, despite substantial reductions since 1990 (Hawaii: −50.61% [−59.02 to −41.20]; DC: −62.19% [−67.92 to −54.67]), remained the states with the highest DALY rates in 2021 (106.00 [89.35–126.37] and 91.58 [77.34–108.83], respectively).

### Burden of GC by sex and age group

In 2021, the age-standardized incidence rate was 6.79 [6.37–7.07] per 100,000 in males and 3.60 [3.27–3.80] in females (eTable 3, eFigure 3). Compared to 1990, these reflect declines from 11.70 [11.17–12.05] in males and 5.31 [4.88–5.55] in females. The narrowing sex gap was due to a greater reduction among men (−42.01% [−44.28 to −39.75]) than women (−32.22% [−34.51 to −29.72]). Similar patterns were observed in mortality (male: −51.59% [–53.53 to –49.67]; female: –42.97% [−44.76 to −40.98]) and DALYs (male: −49.84% [−51.81 to −47.93]; female: −37.68% [−39.66 to −35.56]). Men consistently exhibited higher incidence, mortality, and DALYs across all age groups, and the burden increased with age (eFigure 4).

The proportion of DALYs attributable to risk factors varied by age (eTable 4, eFigure 5). High sodium intake contributed to approximately 7% of DALYs across all strata. Smoking followed a bell-shaped curve, peaking at ages 65–69 in men (25.35% [20.24–30.51]) and women (16.05% [12.56–19.92]). While the sodium-attributable burden remained stable since 1990, the smoking-attributable burden declined substantially across all male age groups and among females up to ages 65–69.

### Burden of GC by SDI, race, and socioeconomic factors

**Figure 2:**
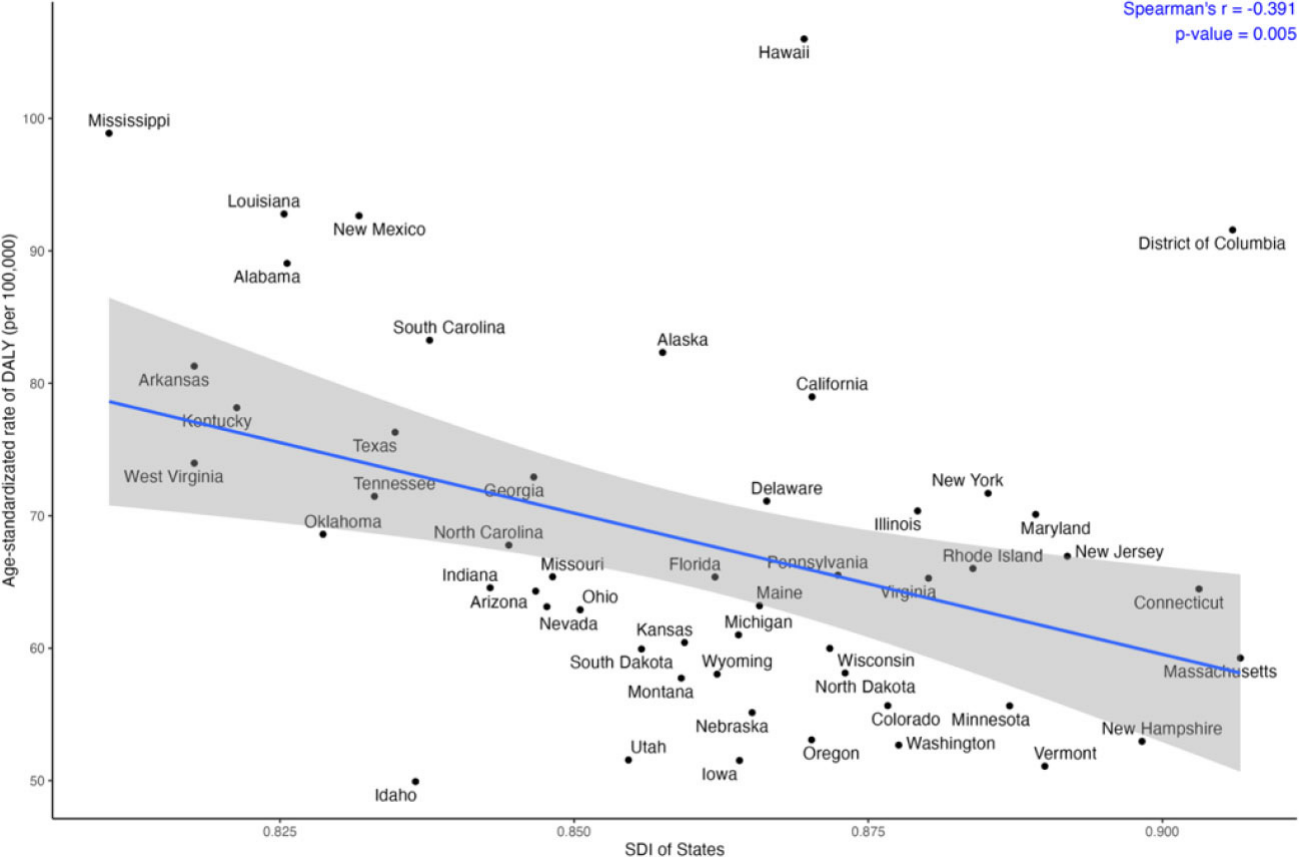
Correlation between SDI and age-standardized DALY rates of gastric cancer in the United States by state, 2021. DALYs = Disability-adjusted life years. Ribbon indicates 95% confidence interval.

Spearman rank tests showed no association between SDI and incidence (ρ = −0.132, *p* = 0.354), but inverse correlations with deaths (ρ = −0.320, *p* = 0.022) and DALYs (ρ = −0.391, *p* = 0.005) (Figure 2, eTable 5, eFigure 6). DALYs were primarily driven by YLLs (*r* = −0.396, *p* = 0.004) rather than YLDs (*r* = −0.047, *p* = 0.743). Hawaii and DC exhibited higher-than-expected mortality and DALY burdens relative to their SDIs.

In multivariate regression, state-level racial/ethnic composition was significantly correlated with all epidemiological measures (*p* < 0.001, Table 2). States with larger Hispanic populations had the lowest burden, while states with larger Asian populations had the highest. These disparities were most pronounced in prevalence (Hispanic: β = 0.145 [0.102–0.187]; Asian: β = 0.628 [0.500–0.755]) and incidence (Hispanic: β = 0.053 [0.039–0.067]; Asian: β = 0.220 [0.178–0.262]), with Asian populations associated with a fourfold higher burden than Hispanic populations. Disparities in YLLs were also evident (Hispanic: β = 0.908 [0.730–1.085]; Asian: β = 2.503 [1.971–3.035]). Because GBD estimates are not race-disaggregated, these represent ecological correlations rather than individual-level risk.

**Table 2:** Multiple regression between incidence, deaths, DALYs, prevalence, YLDs, and YLLs count of gastric cancer and racial/ethnic population, 2021, United States.

MEASURE	HISPANIC		WHITE		BLACK		ASIAN	
	β (95% CI)	*P*-VALUE	β (95% CI)	*P*-VALUE	β (95% CI)	*P*-VALUE	β (95% CI)	*P*-VALUE
**Incidence**	0.053 [0.039, 0.067]	<0.001	0.084 [0.074, 0.094]	<0.001	0.128 [0.099, 0.158]	<0.001	0.220 [0.178, 0.262]	<0.001
**Deaths**	0.032 [0.023, 0.041]	<0.001	0.049 [0.043, 0.056]	<0.001	0.068 [0.049, 0.087]	<0.001	0.122 [0.095, 0.150]	<0.001
**DALYs**	0.925 [0.745, 1.106]	<0.001	0.965 [0.838, 1.092]	<0.001	1.705 [1.324, 2.087]	<0.001	2.568 [2.027, 3.109]	<0.001
**Prevalence**	0.145 [0.102, 0.187]	<0.001	0.219 [0.189, 0.249]	<0.001	0.330 [0.240, 0.420]	<0.001	0.628 [0.500, 0.755]	<0.001
**YLDs**	0.017 [0.013, 0.022]	<0.001	0.026 [0.023, 0.029]	<0.001	0.038 [0.029, 0.048]	<0.001	0.065 [0.052, 0.078]	<0.001
**YLLs**	0.908 [0.730, 1.085]	<0.001	0.939 [0.814, 1.065]	<0.001	1.667 [1.292, 2.042]	<0.001	2.503 [1.971, 3.035]	<0.001

When adjusting for household income and education (Table 3, eTable 8), racial/ethnic composition remained strongly associated with the burden (*p* < 0.001). DALYs were highest in Asian (β = 4.616 [3.200–6.032]) and Black (β = 1.901 [1.479–2.323]) populations, intermediate in White (β = 1.542 [1.150–1.934]), and lowest in Hispanic (β = 1.293 [1.003–1.584]) populations. Income was inversely associated with the burden (β = −34.419 [−60.265 to −8.572], *p* = 0.010), while education showed a positive association (β = 3.097 [0.106–6.087], *p* = 0.043).

**Table 3:** Multiple regression between DALYs of gastric cancer and race/ethnicity and mediation analysis with aggregate household income and educational attainment.

MODEL	VARIABLE	HISPANIC		WHITE		BLACK		ASIAN	
		β (95% CI)	*P*-VALUE	β (95% CI)	*P*-VALUE	β (95% CI)	*P*-VALUE	β (95% CI)	*P*-VALUE
**Multiple regression**	Racial/ethnic population	1.293 [1.003, 1.584]	<0.001	1.542 [1.150, 1.934]	<0.001	1.901 [1.479, 2.323]	<0.001	4.616 [3.200, 6.032]	<0.001
	Aggregate household income	β (95% CI) = −34.419 [−60.265, −8.572] *p*-value = 0.010							
	Education attainment	β (95% CI) = 3.097 [0.106, 6.087] *p*-value = 0.043							
**Mediation analysis**	Total effect	0.925 [0.723, 1.502]	<0.001	0.965 [0.777, 1.098]	<0.001	1.705 [1.327, 2.542]	<0.001	2.568 [−0.062, 3.142]	0.003
	Direct association	1.293 [0.926, 1.987]	<0.001	1.542 [1.028, 1.938]	<0.001	1.901 [1.377, 2.790]	<0.001	4.616 [0.453, 6.096]	<0.001
	Indirect association (combined)	−0.368 [−0.754, 0.014]	0.041	−0.577 [−0.989, −0.023]	0.013	−0.196 [−0.590, 0.192]	0.297	−2.048 [−3.887, 0.087]	0.034
	Indirect association (income)	−0.802 [−1.425, 0.365]	0.067	−1.368 [−2.349, 0.401]	0.052	−1.002 [−2.083, 0.213]	0.084	−4.074 [−8.691, 1.814]	0.121
	Indirect association (education)	0.434 [−0.515, 0.869]	0.215	0.791 [−0.599, 1.544]	0.152	0.807 [−0.430, 1.861]	0.167	2.026 [−2.596, 6.124]	0.32
	Proportion mediated* (%)	22.168		27.223		9.336		30.731	

*To address the opposing signs between indirect and direct effects, we calculated the proportion mediated using the absolute values of the effects.

Mediation analysis showed that while income and education individually had limited indirect effects, their combined indirect associations significantly influenced DALYs (primarily via YLLs) for Hispanic (β = −0.368 [−0.745 to 0.014], *p* = 0.041), White (β = −0.577 [−0.989 to −0.023], *p* = 0.013), and Asian (β = −2.048 [−3.887 to −0.087], *p* = 0.034) populations. The proportion mediated ranged from 22.17% in Hispanics to 30.73% in Asians.

## Discussion

### Uneven progress in US GC burden

This study highlights a dual narrative of substantial national progress and persistent inequities in US GC burden. Consistent with global trends, age-standardized GC mortality declined substantially over the past three decades; however, these gains have been uneven and socially patterned, reflecting broader international observations that improvements in cancer outcomes often accrue disproportionately to advantaged populations. Although age-standardized GC mortality declined by 47% between 1990 and 2021, the gains were not evenly distributed across sociodemographic strata. Racial and ethnic composition and socioeconomic context remained important correlates of the burden: states with larger Asian populations carried the highest DALY rates, followed by those with larger Black, White, and Hispanic populations. These findings are consistent with prior individual-level research showing that Asian [11] and Black [12] populations continue to experience higher GC mortality than White populations in nearly all US counties [13]. Such patterns mirror global incidence gradients, in which GC burden remains concentrated in populations with early-life exposure in high-incidence regions, even as national averages decline. Disparities are further shaped by access to care; for example, Black GC patients in Medicaid-expansion states had a 7.7% lower mortality than their counterparts in non-expansion states.

Race and ethnicity remained significant correlates of state-level burden even after adjustment for income and education, underscoring the multifactorial nature of GC disparities. Interestingly, states with larger Hispanic populations were associated with lower GC burden than those with larger White populations—contrary to prior individual-level studies. This discrepancy is consistent with international ecological analyses showing that area-level measures may obscure within-group heterogeneity driven by nativity, age structure, migration history, and access to diagnosis, and likely reflects the ecological nature of our analysis and broader contextual factors such as healthcare infrastructure, underdiagnosis, and demographic characteristics.

These patterns are consistent with our recent SEER-22 analysis of seven high-risk states, which found gastric adenocarcinoma frequently diagnosed at a distant stage, with survival disparities by race/ethnicity and geography [11]. Together with global evidence linking late-stage diagnosis to socioeconomic and structural barriers, these findings reinforce that inequities in burden are closely tied to inequities in early detection and prevention. Together, these studies highlight that inequities in burden are mirrored by poor outcomes, reinforcing the need for risk-based screening and early detection.

### Geographic and structural disparities

GC burden was the highest in southern states such as Mississippi and New Mexico, where declines since 1990 were modest compared to steep reductions in the northeast (with most states experiencing >50% reductions). Hawaii and DC persisted as high-burden outliers despite substantial long-term declines. These regional patterns resemble global observations in which GC burden is highest in settings characterized by structural disadvantage, limited access to specialized care, and higher prevalence of infection-related risk factors. Structural barriers to care—limited access to tertiary cancer centers, transportation challenges in rural areas, long wait times, insurance gaps, and strained healthcare systems—likely contribute to these patterns [14, 15]. While community-based initiatives (e.g., the Mississippi Partnership for Comprehensive Cancer Control) have begun offering low-cost or no-cost cancer screenings [16], gaps in infrastructure remain large.

Southern and coastal states may also carry a higher *H. pylori* burden, partly due to larger immigrant populations known to have elevated prevalence [8]. This parallels global epidemiologic evidence showing that *H. pylori* prevalence remains highest in low- and middle-income countries and among migrant populations originating from high-incidence regions. Dietary and lifestyle factors, including household crowding, high sodium intake, obesity, and tobacco use, further amplify risk [17, 18]. These interrelated structural, infectious, and behavioral determinants underscore why regional disparities persist despite overall national progress.

### Socioeconomic determinants and modifiable risks

Socioeconomic status emerged as a key driver of disease burden. Mediation analysis demonstrated that income and education together explained up to 30% of racial and ethnic disparities, but large gaps remained, consistent with prior studies linking low socioeconomic position to GC incidence and premature mortality [19]. Similar socioeconomic gradients in GC burden have been documented globally, underscoring that poverty, education, and access to care shape risk and outcomes across diverse health systems.

Among modifiable risks, *H. pylori* infection remains the most established and actionable, yet remains chronically underaddressed in US public health policy. High salt intake has been strongly associated with *H. pylori*-induced gastric carcinogenesis [20], and in this study contributed to approximately 7% of DALYs across all sex–age groups. Smoking-attributable burden, by contrast, declined markedly since 1990, particularly among men, reflecting the impact of public health interventions [21, 22]. These trends are consistent with global patterns, in which tobacco control has reduced GC risk while infection-related and dietary risks persist. These findings align with prior evidence but emphasize that absolute case numbers may still rise due to demographic shifts [23].

A 2014 national survey revealed gaps in physician practice: only 58% of gastroenterologists confirmed *H. pylori* eradication after treatment, and one-third failed to assess prior antibiotic use [24]. Many were also unfamiliar with antibiotic resistance rates. Comparable gaps in *H. pylori* detection and eradication have been documented internationally, particularly in settings without organized screening programs. Together with our findings, this highlights urgent opportunities to improve *H. pylori* management and dietary interventions at the population level.

### Global lessons and missed US opportunities

Globally, age-standardized GC rates have declined, yet absolute burden remains high among minority and socioeconomically disadvantaged populations [25]. This pattern underscores a central lesson for global health: aggregate progress does not equate to equity. The US contrasts starkly with Japan and South Korea, where population-based screening has improved early detection and survival. In these countries, five-year survival rates approach 70%, compared with <30% in most other settings [26, 27]. Advances such as image-enhanced endoscopy and artificial intelligence have further strengthened these programs [28, 29]. These international experiences demonstrate that targeted, risk-based prevention strategies can substantially reduce disparities when implemented at scale. The absence of comparable initiatives in the US—particularly for high-risk immigrant populations—represents a missed opportunity for prevention and equity.

### Limitations

Our analyses are limited by the inability to disaggregate the burden by cancer subtype (cardia versus non-cardia) and by the fact that GBD estimates are not stratified by race or ethnicity. In some cases, limited data availability may have led to the grouping of diverse racial and ethnic populations, further reducing the precision of these estimates. Because our unit of analysis was the state, all inferences are ecological; individual-level conclusions cannot be drawn from these data. As such, our findings linking state-level racial/ethnic composition to GC burden reflect associations at the population level, not direct within-group differences. Interpretation must be cautious, as the observed associations may be influenced by broader demographic, structural, or healthcare system-level factors. Data sources may also underrepresent the true burden of GC, as the lack of national screening and surveillance guidelines in the US may lead to underdiagnosis and later-stage detection [30]. Similarly, the association between educational attainment and GC burden should be interpreted with caution, as it likely reflects broader socioeconomic pathways rather than a direct causal relationship [19].

## Conclusion

Despite overall declines in US GC incidence and mortality, our findings reveal persistent and unevenly distributed burdens across states and populations. These patterns reflect broader global experience, in which progress in GC control has not been equitably shared across socioeconomic or demographic groups. By integrating DALYs with state-level sociodemographic and socioeconomic data, this study demonstrates that racial/ethnic composition, income, and education remain powerful drivers of inequity, with up to one-third of excess burden mediated by structural factors. Viewed in a global health context, the US experience illustrates how exposure-linked risk, migration, and social conditions interact to shape cancer disparities even in high-income settings. These insights highlight the need for targeted, migration- and equity-informed prevention strategies—particularly *H. pylori* screening and eradication and dietary risk reduction—in the communities and states where the burden remains highest.

## Additional File

The additional file for this article can be found as follows:

Supplementary Material 1. List of Supplementary Tables and Figures.
