# Supplementary material for: Beyond Incidence and Mortality: Socioeconomic Mediation of Gastric Cancer Disparities in the United States, 1990–2021
Source: Ann Glob Health. 2026 Feb 4;92(1):14. doi: 10.5334/aogh.5015 (PMC12880004; doi:10.5334/aogh.5015)
Supplement: Supplementary Material 1. — List of Supplementary Tables and Figures. [file agh-92-1-5015-s1.pdf]

Supplementary material for burden of gastric cancer in the United States 1990 – 2021: a systematic analysis of trends and patterns of geographic variations for the global burden of disease study

List of Supplementary Tables and Figures

Appendix I: Supplementary methods ... 3

eTable 1: GATHER checklist ... 3

R session information for data visualization and secondary analysis ... 4

Appendix II: Supplementary tables ... 6

eTable 2a: Number and age-standardized rate of incidence, death, DALY rate of gastric cancer, 2021, and percentage change from 1990 to 2021 ... 6

eTable 2b: Number and age-standardized rate of prevalence, YLD, YLL rate of gastric cancer, 2021, and percentage change from 1990 to 2021 ... 11

eTable 3a: Incidence and death rate of gastric cancer, by age and sex, United States, 2021 ... 16

eTable 3b: DALY and prevalence rate of gastric cancer, by age and sex, United States, 2021 ... 18

eTable 3c: YLD and YLL rate of gastric cancer, by age and sex, United States, 2021 ... 20

eTable 4: Proportion of risk-attributable DALYs for gastric cancer, by age and sex, United States, 2021 ... 22

eTable 8a: Results of secondary statistical analysis, multiple linear regression ... 24

eTable 8b: Results of secondary statistical analysis, mediation analysis, incidence ... 24

eTable 8c: Results of secondary statistical analysis, mediation analysis, deaths ... 25

eTable 8d: Results of secondary statistical analysis, mediation analysis, prevalence ... 25

eTable 8e: Results of secondary statistical analysis, mediation analysis, YLDs ... 26

eTable 8f: Results of secondary statistical analysis, mediation analysis, YLLs ... 26

Appendix II: Supplementary figures ... 27

eFigure 1: Age-standardized rate of (A) prevalence, (B) YLDs, and (C) YLLs rate of gastric cancer, 2021, United States ... 28

eFigure 2: Count and age-standardized rate of (A) incidence, (B) death, (C) DALY, (D) prevalence, (E) YLD, and (F) YLL of gastric cancer, 1990-2021, United States ... 27

eFigure 3: Age-standardized rate of (A) incidence, (B) death, (C) DALY, (D) prevalence, (E) YLD, and (F) YLL of gastric cancer, male and female, 1990-2021, United States ... 29

eFigure 4: Rate of (A) incidence, (B) death, (C) DALY, (D) prevalence, (E) YLD, and (F) YLL of gastric cancer by age-group, male and female, 2021, United States ... 30

eFigure 5: Proportion of risk-attributable DALYs of Gastric Cancer, by age and sex, United States, 2021 ... 31

eFigure 6: Correlation between SDI and age-standardized rates of (A) incidence (B) death (C) prevalence (D) YLDs (E) YLLs for gastric cancer by state, 2021, United States ... 32

Appendix I: Supplementary methods

eTable 1: GATHER checklist

#	Checklist item	Section/paragraph/ interpretation
**Objectives and funding**		
1	Define the indicators, populations, and time periods for which estimates were made.	Methods / "Overview" section
2	List the funding sources for the work.	No funding
**Data inputs**		
For all data inputs from multiple sources that are synthesized as part of the study:		
3	Describe how the data were identified and how the data were accessed.	Methods / "Data Sources and Case Definition" section, the details have been published previously.
4	Specify the inclusion and exclusion criteria. Identify all ad-hoc exclusions.	Methods / "Data Sources and Case Definition" section
5	Provide information on all included data sources and their main characteristics. For each data source used, report reference information or contact name/institution, population represented, data collection method, year(s) of data collection, sex and age range, diagnostic criteria or measurement method, and sample size, as relevant.	Available via online data source tools (http://ghdx.healthdata.org/gbd-2021/data-input-sources).
6	Identify and describe any categories of input data that have potentially important biases (e.g., based on characteristics listed in item 5).	Methods / "Data Sources and Case Definition" sections, the details have been published previously.
For data inputs that contribute to the analysis but were not synthesized as part of the study:		
7	Describe and give sources for any other data inputs.	Available via online data source tools (http://ghdx.healthdata.org/gbd-2021/data-input-sources).
For all data inputs:		
8	Provide all data inputs in a file format from which data can be efficiently extracted (e.g., a spreadsheet as opposed to a PDF), including all relevant meta-data listed in item 5. For any data inputs that cannot be shared due to ethical or legal reasons, such as third-party ownership, provide a contact name or the name of the institution that retains the right to the data.	Available via online data source tools (http://ghdx.healthdata.org/gbd-2021/data-input-sources); input data not available in tools will be made available upon request.
**Data analysis**		
9	Provide a conceptual overview of the data analysis method. A diagram may be helpful.	Flow diagrams of the overall methodological processes were available online (http://ghdx.healthdata.org/gbd-2021/code)
10	Provide a detailed description of all steps of the analysis, including mathematical formulae. This description should cover, as relevant, data cleaning, data pre-processing, data adjustments and weighting of data sources, and mathematical or statistical model(s).	Methods / "Estimation of Fatal and Non-fatal Outcomes" section, the details have been published previously.
11	Describe how candidate models were evaluated and how the final model(s) were selected.	Methods / "Estimation of Fatal and Non-fatal Outcomes" section, the details have been published previously.
12	Provide the results of an evaluation of model performance, if done, as well as the results of any relevant sensitivity analysis.	Methods / "Estimation of Fatal and Non-fatal Outcomes" section, the details have been published previously.
13	Describe methods for calculating uncertainty of the estimates. State which sources of uncertainty were, and were not, accounted for in the uncertainty analysis.	Methods / "Statistical Analysis" section, the details have been published previously.
14	State how analytic or statistical source code used to generate estimates can be accessed.	Methods / "Statistical Analysis" section
**Results and discussion**		
15	Provide published estimates in a file format from which data can be efficiently extracted.	Results, and online data tools (data visualization tools, and data query tools, http://ghdx.healthdata.org/gbd-2021)
16	Report a quantitative measure of the uncertainty of the estimates (e.g. uncertainty intervals).	Results, and online data tools (data visualization tools, and data query tools, http://ghdx.healthdata.org/gbd-2021)
17	Interpret results in light of existing evidence. If updating a previous set of estimates, describe the reasons for changes in estimates.	Discussion
18	Discuss limitations of the estimates. Include a discussion of any modelling assumptions or data limitations that affect interpretation of the estimates.	Discussion

R session information for data visualization and secondary analysis

```
> sessionInfo()
R version 4.5.1 (2025-06-13)
Platform: aarch64-apple-darwin20
Running under: macOS Sequoia 15.6.1

Matrix products: default
BLAS:
/System/Library/Frameworks/Accelerate.framework/Versions/A/Frameworks/vecLib.framewor
k/Versions/A/libBLAS.dylib
LAPACK: /Library/Frameworks/R.framework/Versions/4.5-
arm64/Resources/lib/libRlapack.dylib;  LAPACK version 3.12.1

locale:
[1] en_US.UTF-8/en_US.UTF-8/en_US.UTF-8/C/en_US.UTF-8/en_US.UTF-8

time zone: Asia/Seoul
tzcode source: internal

attached base packages:
[1] stats     graphics  grDevices utils     datasets  methods   base

other attached packages:
 [1] lavaan_0.6-19     broom_1.0.9       usmap_1.0.0       ggpmisc_0.6.2
 [5] ggpp_0.5.9        ggrepel_0.9.6     cowplot_1.2.0     scales_1.4.0
 [9] patchwork_1.3.2   data.table_1.17.8 lubridate_1.9.4   forcats_1.0.0
[13] stringr_1.5.1     dplyr_1.1.4       purrr_1.1.0       readr_2.1.5
[17] tidyr_1.3.1       tibble_3.3.0      ggplot2_3.5.2     tidyverse_2.0.0

loaded via a namespace (and not attached):
 [1] generics_0.1.4     stringi_1.8.7      lattice_0.22-7     hms_1.1.3
 [5] digest_0.6.37      magrittr_2.0.3     evaluate_1.0.5     grid_4.5.1
```

```
 [9] timechange_0.3.0   RColorBrewer_1.1-3 fastmap_1.2.0      Matrix_1.7-3
[13] backports_1.5.0    survival_3.8-3     pbivnorm_0.6.0     mnormt_2.1.1
[17] cli_3.6.5          rlang_1.1.6        splines_4.5.1      withr_3.0.2
[21] yaml_2.3.10        tools_4.5.1        MatrixModels_0.5-4 SparseM_1.84-2
[25] polynom_1.4-1      tzdb_0.5.0         vctrs_0.6.5        R6_2.6.1
[29] stats4_4.5.1       lifecycle_1.0.4    MASS_7.3-65        pkgconfig_2.0.3
[33] pillar_1.11.0      gtable_0.3.6       glue_1.8.0         Rcpp_1.1.0
[37] xfun_0.53          tidyselect_1.2.1   rstudioapi_0.17.1  knitr_1.50
[41] farver_2.1.2       htmltools_0.5.8.1  rmarkdown_2.29     compiler_4.5.1
[45] quadprog_1.5-8     quantreg_6.1
```

Appendix II: Supplementary tables

eTable 2a: Number and age-standardized rate of incidence, death, DALY rate of gastric cancer, 2021, and percentage change from 1990 to 2021

	Incidence (95% UI)			Deaths (95% UI)			DALYs (95% UI)		
	**Absolute number, thousands, 2021**	**Age-standardized rate, per 100,000 people, 2021**	**Percentage Change, 1990-2021**	**Absolute number, thousands, 2021**	**Age-standardized rate, per 100,000 people, 2021**	**Percentage Change, 1990-2021**	**Absolute number, thousands, 2021**	**Age-standardized rate, per 100,000 people, 2021**	**Percentage Change, 1990-2021**
**United States of America**	28.46 [26.39–29.87]	5.07 [4.74–5.30]	-36.97% [-38.82% to -35.11%]	16.44 [15.03–17.35]	2.84 [2.62–2.98]	-47.04% [-48.71% to -45.33%]	363.14 [342.10–377.92]	69.16 [65.84–71.77]	-44.63% [-46.23% to -43.02%]
**Alabama**	0.48 [0.42–0.56]	5.69 [4.88–6.57]	-30.47% [-41.07% to -19.49%]	0.31 [0.27–0.36]	3.57 [3.08–4.14]	-38.26% [-47.20% to -28.41%]	7.09 [6.08–8.24]	89.06 [76.22–103.28]	-35.27% [-44.95% to -25.10%]
**Alaska**	0.06 [0.05–0.07]	6.01 [5.14–6.93]	-38.37% [-47.13% to -28.11%]	0.03 [0.03–0.04]	3.35 [2.86–3.89]	-50.24% [-56.89% to -42.68%]	0.82 [0.72–0.95]	82.33 [71.72–95.10]	-46.76% [-53.91% to -38.51%]
**Arizona**	0.58 [0.50–0.69]	4.74 [4.03–5.56]	-33.04% [-44.26% to -21.44%]	0.33 [0.28–0.39]	2.60 [2.21–3.04]	-43.81% [-52.15% to -34.06%]	7.31 [6.23–8.58]	64.31 [54.57–75.06]	-41.04% [-50.59% to -29.98%]
**Arkansas**	0.26 [0.22–0.31]	5.21 [4.45–6.09]	-30.41% [-41.59% to -17.83%]	0.17 [0.14–0.19]	3.22 [2.75–3.74]	-38.13% [-47.22% to -27.22%]	3.80 [3.23–4.42]	81.30 [69.12–94.83]	-34.26% [-44.70% to -22.18%]
**California**	3.50 [2.95–4.06]	5.53 [4.66–6.40]	-27.02% [-38.14% to -14.93%]	2.04 [1.72–2.35]	3.16 [2.68–3.64]	-42.40% [-50.97% to -33.47%]	46.73 [39.51–53.72]	78.98 [66.99–90.83]	-38.61% [-47.88% to -28.51%]
**Colorado**	0.41 [0.33–0.48]	4.35 [3.57–5.20]	-39.42% [-49.52% to -27.89%]	0.22 [0.18–0.27]	2.37 [1.95–2.82]	-48.62% [-57.24% to -38.90%]	4.94 [4.08–5.92]	55.66 [45.90–66.27]	-47.34% [-56.63% to -37.36%]
**Connecticut**	0.33 [0.28–0.39]	4.98 [4.14–5.88]	-45.32% [-55.93% to -34.36%]	0.19 [0.16–0.23]	2.70 [2.25–3.18]	-54.75% [-62.89% to -46.47%]	4.01 [3.34–4.75]	64.47 [53.19–76.17]	-52.68% [-61.39% to -43.41%]

**Delaware**	0.09 [0.08–0.11]	5.18 [4.39–5.99]	-35.42% [-44.84% to -25.88%]	0.06 [0.05–0.06]	2.90 [2.49–3.32]	-46.83% [-54.20% to -39.23%]	1.20 [1.03–1.38]	71.11 [61.60–81.64]	-43.81% [-51.77% to -35.38%]
**District of Columbia**	0.06 [0.05–0.07]	5.77 [4.88–6.84]	-48.58% [-56.35% to -38.42%]	0.04 [0.03–0.04]	3.66 [3.04–4.36]	-60.33% [-66.35% to -52.69%]	0.85 [0.72–1.01]	91.58 [77.34–108.83]	-62.19% [-67.92% to -54.67%]
**Florida**	1.93 [1.58–2.32]	4.69 [3.86–5.64]	-35.74% [-46.00% to -23.73%]	1.13 [0.94–1.34]	2.59 [2.14–3.06]	-45.96% [-53.54% to -36.68%]	24.68 [20.49–29.29]	65.38 [54.32–77.35]	-42.60% [-51.11% to -31.91%]
**Georgia**	0.97 [0.82–1.12]	5.86 [4.94–6.77]	-33.40% [-44.12% to -23.03%]	0.51 [0.43–0.58]	3.02 [2.56–3.46]	-45.92% [-54.18% to -37.74%]	11.70 [9.97–13.55]	72.92 [62.20–84.60]	-45.76% [-54.18% to -36.55%]
**Hawaii**	0.21 [0.18–0.25]	7.88 [6.65–9.29]	-45.88% [-54.37% to -35.95%]	0.13 [0.10–0.15]	4.31 [3.58–5.12]	-54.51% [-61.62% to -46.37%]	2.62 [2.21–3.11]	106.00 [89.35–126.37]	-50.61% [-59.02% to -41.20%]
**Idaho**	0.11 [0.10–0.14]	3.82 [3.20–4.46]	-36.65% [-46.92% to -25.69%]	0.07 [0.06–0.08]	2.19 [1.83–2.54]	-45.35% [-54.21% to -36.48%]	1.41 [1.18–1.65]	49.93 [42.12–58.06]	-44.45% [-54.09% to -35.43%]
**Illinois**	1.14 [0.94–1.34]	5.28 [4.38–6.24]	-37.31% [-47.50% to -25.53%]	0.65 [0.54–0.76]	2.91 [2.45–3.42]	-49.46% [-56.97% to -40.49%]	14.22 [11.91–16.74]	70.36 [59.09–82.81]	-47.17% [-55.54% to -37.48%]
**Indiana**	0.51 [0.42–0.59]	4.52 [3.79–5.28]	-30.41% [-40.85% to -19.02%]	0.30 [0.25–0.35]	2.64 [2.22–3.06]	-38.39% [-47.22% to -28.74%]	6.77 [5.69–7.91]	64.56 [54.22–75.38]	-34.85% [-44.51% to -23.54%]
**Iowa**	0.23 [0.19–0.28]	4.10 [3.37–4.86]	-31.22% [-43.98% to -18.12%]	0.13 [0.11–0.16]	2.17 [1.78–2.59]	-38.26% [-49.25% to -27.11%]	2.73 [2.24–3.26]	51.52 [42.26–61.36]	-35.66% [-47.40% to -23.73%]
**Kansas**	0.22 [0.18–0.26]	4.51 [3.72–5.42]	-30.49% [-41.87% to -16.64%]	0.13 [0.11–0.15]	2.53 [2.10–2.99]	-37.87% [-47.55% to -26.26%]	2.74 [2.29–3.24]	60.43 [50.18–71.71]	-36.19% [-46.89% to -23.88%]
**Kentucky**	0.42 [0.36–0.51]	5.60 [4.74–6.66]	-30.06% [-41.31% to -17.80%]	0.25 [0.21–0.29]	3.17 [2.67–3.78]	-35.89% [-46.18% to -24.99%]	5.55 [4.69–6.67]	78.16 [66.04–93.42]	-32.84% [-44.14% to -20.56%]
**Louisiana**	0.49 [0.41–0.58]	6.70 [5.67–7.93]	-33.68% [-43.88% to -21.40%]	0.28 [0.23–0.32]	3.69 [3.13–4.30]	-42.25% [-51.38% to -32.11%]	6.43 [5.46–7.56]	92.79 [78.76–109.36]	-39.11% [-49.02% to -27.60%]
**Maine**	0.13 [0.11–0.16]	4.73 [3.96–5.60]	-38.28% [-48.78% to -26.18%]	0.08 [0.07–0.09]	2.64 [2.21–3.12]	-45.77% [-54.29% to -35.89%]	1.63 [1.36–1.93]	63.20 [52.74–74.80]	-42.33% [-52.03% to -30.74%]

**Maryland**	0.53 [0.44–0.62]	5.07 [4.22–6.00]	-41.16% [-50.87% to -29.82%]	0.31 [0.25–0.36]	2.88 [2.37–3.37]	-51.27% [-59.41% to -42.47%]	6.86 [5.69–8.15]	70.10 [58.24–83.25]	-48.53% [-57.26% to -38.61%]
**Massachusetts**	0.63 [0.51–0.75]	4.97 [4.07–5.87]	-49.52% [-58.25% to -40.29%]	0.35 [0.28–0.41]	2.61 [2.13–3.07]	-57.38% [-64.70% to -49.67%]	7.06 [5.77–8.26]	59.26 [48.72–69.21]	-57.06% [-64.75% to -48.82%]
**Michigan**	0.84 [0.70–1.00]	4.62 [3.85–5.47]	-42.70% [-52.39% to -32.20%]	0.48 [0.40–0.56]	2.53 [2.12–2.96]	-50.74% [-58.55% to -42.34%]	10.39 [8.78–12.19]	61.00 [51.59–71.45]	-48.46% [-57.03% to -39.16%]
**Minnesota**	0.47 [0.38–0.56]	4.69 [3.81–5.61]	-38.45% [-48.62% to -26.37%]	0.26 [0.21–0.31]	2.47 [2.03–2.95]	-45.73% [-54.04% to -35.71%]	5.23 [4.32–6.27]	55.64 [45.94–66.54]	-45.08% [-54.17% to -34.47%]
**Mississippi**	0.29 [0.24–0.35]	6.05 [5.09–7.22]	-26.71% [-39.74% to -10.75%]	0.19 [0.16–0.23]	3.92 [3.32–4.66]	-34.55% [-45.50% to -21.98%]	4.45 [3.74–5.30]	98.88 [83.42–117.69]	-30.72% [-42.12% to -16.69%]
**Missouri**	0.50 [0.42–0.59]	4.66 [3.93–5.48]	-28.91% [-40.36% to -14.87%]	0.30 [0.25–0.35]	2.68 [2.26–3.14]	-38.53% [-48.29% to -26.64%]	6.54 [5.51–7.66]	65.39 [55.01–76.91]	-35.55% [-46.50% to -22.60%]
**Montana**	0.09 [0.07–0.10]	4.21 [3.52–5.03]	-36.32% [-46.99% to -23.54%]	0.05 [0.04–0.06]	2.43 [2.05–2.87]	-44.46% [-53.35% to -33.88%]	1.09 [0.92–1.30]	57.74 [48.77–68.54]	-41.67% [-51.39% to -30.16%]
**Nebraska**	0.14 [0.12–0.17]	4.31 [3.59–5.09]	-35.35% [-45.44% to -22.87%]	0.08 [0.07–0.10]	2.36 [1.97–2.79]	-43.96% [-52.55% to -33.38%]	1.69 [1.41–2.00]	55.13 [45.79–65.48]	-42.46% [-51.74% to -31.42%]
**Nevada**	0.23 [0.19–0.27]	4.58 [3.84–5.40]	-40.74% [-50.60% to -30.07%]	0.13 [0.11–0.15]	2.62 [2.20–3.05]	-50.54% [-58.38% to -42.39%]	3.02 [2.52–3.52]	63.13 [52.67–73.77]	-48.83% [-57.45% to -40.28%]
**New Hampshire**	0.12 [0.10–0.14]	4.32 [3.60–5.16]	-43.43% [-53.02% to -32.39%]	0.06 [0.05–0.08]	2.34 [1.95–2.79]	-51.49% [-59.03% to -42.60%]	1.33 [1.10–1.59]	52.97 [44.38–63.40]	-50.89% [-58.77% to -41.20%]
**New Jersey**	0.91 [0.75–1.08]	5.60 [4.61–6.67]	-48.88% [-57.85% to -39.86%]	0.49 [0.40–0.58]	2.87 [2.37–3.39]	-57.05% [-64.16% to -49.43%]	10.27 [8.44–12.16]	66.94 [55.03–79.53]	-56.22% [-63.81% to -47.75%]
**New Mexico**	0.22 [0.19–0.26]	6.02 [5.09–7.09]	-27.53% [-39.58% to -13.92%]	0.13 [0.11–0.16]	3.54 [2.96–4.16]	-38.74% [-48.48% to -27.80%]	3.07 [2.58–3.60]	92.65 [77.82–108.62]	-32.49% [-43.68% to -20.36%]
**New York**	1.94 [1.59–2.28]	5.54 [4.56–6.47]	-39.12% [-49.19% to -29.23%]	1.08 [0.88–1.27]	2.95 [2.43–3.46]	-52.88% [-60.09% to -45.55%]	23.40 [19.47–27.40]	71.69 [60.23–83.68]	-51.61% [-59.40% to -43.60%]

**North Carolina**	0.86 [0.72–1.00]	4.81 [4.02–5.60]	-33.75% [-43.52% to -22.94%]	0.51 [0.43–0.59]	2.79 [2.34–3.22]	-44.64% [-52.26% to -35.62%]	11.28 [9.42–13.09]	67.78 [57.10–79.19]	-42.89% [-51.38% to -33.30%]
**North Dakota**	0.06 [0.05–0.07]	4.26 [3.54–4.98]	-46.11% [-54.98% to -35.81%]	0.03 [0.03–0.04]	2.40 [1.99–2.81]	-52.56% [-60.04% to -43.91%]	0.70 [0.59–0.83]	58.12 [48.64–68.04]	-48.95% [-56.92% to -39.39%]
**Ohio**	0.94 [0.79–1.09]	4.46 [3.77–5.16]	-41.08% [-49.76% to -31.46%]	0.57 [0.48–0.66]	2.63 [2.22–3.04]	-47.92% [-55.62% to -39.80%]	12.23 [10.32–14.20]	62.90 [52.94–73.07]	-45.39% [-53.79% to -36.76%]
**Oklahoma**	0.29 [0.25–0.34]	4.52 [3.86–5.30]	-35.04% [-45.32% to -23.32%]	0.18 [0.16–0.21]	2.78 [2.37–3.25]	-39.74% [-49.02% to -29.32%]	4.10 [3.51–4.80]	68.60 [58.75–80.38]	-37.06% [-46.98% to -24.99%]
**Oregon**	0.33 [0.27–0.39]	4.17 [3.47–5.01]	-37.72% [-48.41% to -26.91%]	0.18 [0.15–0.21]	2.20 [1.83–2.63]	-47.80% [-56.48% to -38.53%]	3.87 [3.21–4.63]	53.08 [44.01–63.63]	-44.82% [-54.28% to -34.83%]
**Pennsylvania**	1.22 [1.01–1.43]	4.94 [4.11–5.79]	-45.02% [-54.13% to -34.84%]	0.71 [0.60–0.84]	2.78 [2.32–3.26]	-53.39% [-60.92% to -45.35%]	14.80 [12.34–17.33]	65.52 [54.42–76.75]	-51.71% [-59.93% to -43.13%]
**Rhode Island**	0.11 [0.09–0.13]	5.35 [4.48–6.32]	-46.81% [-55.49% to -37.54%]	0.06 [0.05–0.07]	2.93 [2.46–3.47]	-54.96% [-61.94% to -47.64%]	1.27 [1.07–1.52]	66.02 [55.51–78.89]	-55.91% [-62.98% to -48.02%]
**South Carolina**	0.49 [0.40–0.57]	5.51 [4.55–6.45]	-30.62% [-41.89% to -18.04%]	0.30 [0.25–0.35]	3.31 [2.77–3.84]	-42.44% [-51.67% to -31.97%]	6.87 [5.72–8.05]	83.25 [69.21–98.30]	-40.30% [-49.81% to -29.30%]
**South Dakota**	0.07 [0.06–0.08]	4.23 [3.58–4.91]	-37.57% [-46.60% to -26.50%]	0.04 [0.03–0.05]	2.46 [2.07–2.86]	-45.17% [-52.81% to -35.59%]	0.84 [0.71–0.97]	59.94 [51.44–69.07]	-41.32% [-49.40% to -30.92%]
**Tennessee**	0.56 [0.47–0.65]	4.77 [4.05–5.58]	-30.48% [-40.64% to -17.76%]	0.34 [0.29–0.40]	2.85 [2.45–3.34]	-39.97% [-48.61% to -29.86%]	7.80 [6.67–9.17]	71.46 [60.77–83.93]	-36.42% [-46.21% to -24.71%]
**Texas**	2.28 [1.95–2.64]	5.47 [4.68–6.31]	-32.15% [-41.91% to -20.72%]	1.30 [1.12–1.49]	3.09 [2.64–3.53]	-42.79% [-50.49% to -34.20%]	30.85 [26.39–35.37]	76.30 [65.40–87.25]	-40.84% [-49.26% to -31.28%]
**Utah**	0.16 [0.14–0.19]	4.03 [3.40–4.69]	-33.93% [-43.53% to -22.77%]	0.09 [0.08–0.11]	2.29 [1.93–2.66]	-43.90% [-51.74% to -35.56%]	2.05 [1.73–2.38]	51.56 [43.53–59.74]	-43.08% [-51.68% to -33.46%]
**Vermont**	0.05 [0.04–0.06]	3.96 [3.44–4.62]	-40.60% [-48.93% to -31.16%]	0.03 [0.03–0.03]	2.16 [1.88–2.50]	-51.53% [-57.82% to -44.09%]	0.60 [0.52–0.70]	51.09 [44.65–59.36]	-49.10% [-55.96% to -40.80%]

**Virginia**	0.73 [0.61–0.85]	5.02 [4.20–5.86]	-35.49% [-45.76% to -24.71%]	0.41 [0.34–0.48]	2.75 [2.32–3.23]	-46.53% [-54.96% to -38.10%]	8.92 [7.50–10.47]	65.28 [54.97–76.54]	-45.30% [-54.50% to -35.66%]
**Washington**	0.56 [0.47–0.66]	4.29 [3.56–5.04]	-42.13% [-51.08% to -31.68%]	0.30 [0.25–0.36]	2.24 [1.88–2.63]	-52.02% [-59.45% to -43.14%]	6.56 [5.48–7.69]	52.68 [43.84–61.48]	-50.77% [-58.81% to -41.57%]
**West Virginia**	0.16 [0.14–0.19]	4.76 [4.02–5.59]	-31.88% [-42.56% to -19.94%]	0.10 [0.09–0.12]	2.89 [2.45–3.40]	-40.04% [-49.27% to -29.52%]	2.27 [1.92–2.69]	73.97 [62.65–87.21]	-33.42% [-44.03% to -20.77%]
**Wisconsin**	0.50 [0.42–0.61]	4.72 [3.90–5.68]	-41.89% [-52.25% to -30.66%]	0.29 [0.24–0.34]	2.58 [2.13–3.10]	-49.02% [-57.27% to -39.93%]	5.95 [4.95–7.18]	59.98 [49.83–72.22]	-46.69% [-56.14% to -36.50%]
**Wyoming**	0.04 [0.04–0.05]	4.25 [3.62–4.91]	-33.89% [-43.35% to -23.23%]	0.02 [0.02–0.03]	2.42 [2.05–2.78]	-44.05% [-51.64% to -35.36%]	0.53 [0.46–0.62]	58.04 [49.97–66.60]	-40.26% [-48.63% to -30.94%]

eTable 2b: Number and age-standardized rate of prevalence, YLD, YLL rate of gastric cancer, 2021, and percentage change from 1990 to 2021

	Prevalence (95% UI)			YLDs (95% UI)			YLLs (95% UI)		
	**Absolute number, thousands, 2021**	**Age-standardized rate, per 100,000 people, 2021**	**Percentage Change, 1990-2021**	**Absolute number, thousands, 2021**	**Age-standardized rate, per 100,000 people, 2021**	**Percentage Change, 1990-2021**	**Absolute number, thousands, 2021**	**Age-standardized rate, per 100,000 people, 2021**	**Percentage Change, 1990-2021**
**United States of America**	74.99 [70.46–78.36]	13.88 [13.12–14.47]	-22.53% [-25.03% to -20.14%]	8.70 [6.43–11.19]	1.59 [1.17–2.05]	-30.40% [-33.78% to -26.97%]	354.44 [333.70–368.71]	67.58 [64.10–70.09]	-44.89% [-46.47% to -43.30%]
**Alabama**	1.12 [0.95–1.30]	13.62 [11.56–15.89]	-18.97% [-31.23% to -3.68%]	0.14 [0.10–0.19]	1.69 [1.17–2.28]	-24.80% [-39.07% to -8.87%]	6.95 [5.95–8.07]	87.36 [74.82–101.16]	-35.44% [-45.07% to -25.29%]
**Alaska**	0.17 [0.14–0.19]	16.28 [13.89–18.83]	-20.30% [-32.28% to -5.19%]	0.02 [0.01–0.02]	1.86 [1.34–2.46]	-30.40% [-42.24% to -15.28%]	0.80 [0.70–0.93]	80.47 [70.04–93.30]	-47.05% [-54.14% to -38.82%]
**Arizona**	1.57 [1.32–1.87]	13.24 [11.20–15.61]	-18.22% [-33.02% to -1.86%]	0.18 [0.12–0.24]	1.50 [1.05–2.01]	-26.84% [-39.78% to -11.90%]	7.13 [6.07–8.35]	62.81 [53.34–73.24]	-41.32% [-50.85% to -30.28%]
**Arkansas**	0.61 [0.51–0.72]	12.73 [10.75–14.98]	-19.23% [-32.42% to -3.62%]	0.08 [0.05–0.10]	1.56 [1.09–2.11]	-25.80% [-39.35% to -10.62%]	3.72 [3.16–4.34]	79.73 [67.63–93.33]	-34.41% [-44.77% to -22.32%]
**California**	9.43 [7.89–11.00]	15.26 [12.88–17.76]	-6.36% [-21.87% to 10.45%]	1.07 [0.75–1.46]	1.72 [1.20–2.34]	-18.66% [-32.94% to -1.71%]	45.66 [38.69–52.35]	77.25 [65.53–88.77]	-38.94% [-48.18% to -28.99%]
**Colorado**	1.11 [0.91–1.35]	12.21 [10.07–14.71]	-26.80% [-39.78% to -10.68%]	0.13 [0.09–0.18]	1.38 [0.95–1.93]	-33.42% [-46.63% to -17.16%]	4.82 [3.98–5.76]	54.28 [44.84–64.56]	-47.62% [-56.87% to -37.61%]
**Connecticut**	0.89 [0.73–1.05]	14.22 [11.74–16.78]	-31.88% [-45.15% to -17.86%]	0.10 [0.07–0.14]	1.59 [1.09–2.21]	-38.68% [-51.08% to -24.02%]	3.91 [3.23–4.61]	62.88 [51.92–74.13]	-52.95% [-61.65% to -43.75%]
**Delaware**	0.25 [0.21–0.29]	14.04 [11.92–16.47]	-17.96% [-30.54% to -3.88%]	0.03 [0.02–0.04]	1.61 [1.13–2.22]	-27.49% [-41.33% to -10.99%]	1.17 [1.00–1.34]	69.50 [60.26–79.74]	-44.10% [-52.00% to -35.73%]

**District of Columbia**	0.13 [0.11–0.15]	13.56 [11.27–16.20]	-27.79% [-39.44% to -13.22%]	0.02 [0.01–0.02]	1.70 [1.21–2.37]	-41.15% [-52.22% to -27.24%]	0.84 [0.71–0.99]	89.87 [75.80–106.92]	-62.45% [-68.14% to -54.95%]
**Florida**	5.09 [4.20–6.20]	13.59 [11.29–16.46]	-21.90% [-34.67% to -5.94%]	0.60 [0.41–0.82]	1.53 [1.04–2.09]	-28.56% [-41.87% to -12.58%]	24.08 [19.98–28.52]	63.85 [53.32–75.63]	-42.87% [-51.28% to -32.17%]
**Georgia**	2.60 [2.19–3.03]	15.94 [13.47–18.56]	-11.04% [-25.95% to 4.27%]	0.30 [0.21–0.40]	1.82 [1.25–2.45]	-25.35% [-39.83% to -9.46%]	11.40 [9.74–13.18]	71.10 [60.59–82.34]	-46.13% [-54.53% to -37.04%]
**Hawaii**	0.57 [0.47–0.67]	22.45 [18.75–26.55]	-34.22% [-44.93% to -21.97%]	0.06 [0.05–0.09]	2.46 [1.74–3.33]	-39.96% [-52.04% to -26.01%]	2.56 [2.16–3.03]	103.54 [87.34–123.41]	-50.82% [-59.23% to -41.45%]
**Idaho**	0.30 [0.24–0.35]	10.21 [8.45–11.89]	-24.57% [-37.83% to -9.91%]	0.04 [0.02–0.05]	1.19 [0.84–1.66]	-30.75% [-45.11% to -12.47%]	1.37 [1.15–1.60]	48.74 [41.10–56.61]	-44.72% [-54.38% to -35.70%]
**Illinois**	3.03 [2.52–3.64]	14.66 [12.16–17.63]	-18.64% [-31.81% to -1.74%]	0.35 [0.24–0.48]	1.66 [1.15–2.27]	-29.16% [-42.23% to -13.83%]	13.87 [11.63–16.34]	68.71 [57.58–80.75]	-47.49% [-55.86% to -37.88%]
**Indiana**	1.27 [1.05–1.49]	11.74 [9.74–13.77]	-19.57% [-32.56% to -5.98%]	0.15 [0.11–0.21]	1.39 [0.97–1.90]	-25.07% [-39.28% to -8.29%]	6.62 [5.59–7.72]	63.17 [53.21–73.76]	-35.04% [-44.62% to -23.63%]
**Iowa**	0.64 [0.52–0.76]	11.72 [9.55–13.92]	-21.28% [-36.83% to -5.31%]	0.07 [0.05–0.10]	1.32 [0.92–1.86]	-25.63% [-40.11% to -9.02%]	2.65 [2.18–3.17]	50.20 [41.31–59.82]	-35.88% [-47.70% to -23.92%]
**Kansas**	0.57 [0.47–0.68]	12.23 [10.02–14.65]	-20.90% [-35.21% to -3.47%]	0.07 [0.05–0.09]	1.42 [0.97–1.92]	-25.86% [-41.06% to -6.80%]	2.68 [2.23–3.17]	59.01 [49.03–69.95]	-36.40% [-47.01% to -24.31%]
**Kentucky**	1.13 [0.95–1.34]	15.53 [13.12–18.42]	-21.84% [-35.58% to -6.12%]	0.13 [0.09–0.18]	1.75 [1.23–2.39]	-26.90% [-40.61% to -10.59%]	5.42 [4.59–6.49]	76.41 [64.43–91.05]	-32.97% [-44.27% to -20.73%]
**Louisiana**	1.27 [1.05–1.51]	17.93 [14.97–21.33]	-22.23% [-35.34% to -6.88%]	0.15 [0.10–0.20]	2.06 [1.46–2.80]	-27.98% [-41.40% to -11.48%]	6.28 [5.33–7.40]	90.72 [76.98–106.83]	-39.32% [-49.28% to -27.78%]
**Maine**	0.35 [0.29–0.41]	12.87 [10.63–15.30]	-28.46% [-41.36% to -14.01%]	0.04 [0.03–0.06]	1.48 [1.04–2.06]	-32.79% [-45.51% to -17.25%]	1.59 [1.33–1.89]	61.72 [51.57–73.22]	-42.53% [-52.24% to -31.05%]
**Maryland**	1.36 [1.12–1.63]	13.59 [11.24–16.21]	-25.52% [-38.25% to -10.43%]	0.16 [0.11–0.22]	1.57 [1.12–2.14]	-34.24% [-47.59% to -18.11%]	6.70 [5.55–7.96]	68.53 [56.70–81.33]	-48.79% [-57.60% to -38.93%]

**Massachusetts**	1.77 [1.44–2.11]	14.48 [11.81–17.24]	-39.10% [-50.55% to -26.81%]	0.19 [0.13–0.27]	1.57 [1.04–2.14]	-44.36% [-56.35% to -31.95%]	6.87 [5.62–8.04]	57.69 [47.43–67.43]	-57.32% [-64.94% to -49.20%]
**Michigan**	2.26 [1.86–2.72]	12.94 [10.69–15.53]	-31.50% [-44.29% to -17.28%]	0.26 [0.18–0.37]	1.47 [1.04–2.05]	-36.89% [-49.13% to -21.49%]	10.13 [8.54–11.87]	59.53 [50.25–69.62]	-48.69% [-57.20% to -39.48%]
**Minnesota**	1.31 [1.08–1.59]	13.65 [11.23–16.51]	-28.95% [-41.35% to -14.57%]	0.15 [0.10–0.20]	1.50 [1.02–2.02]	-33.20% [-46.00% to -17.62%]	5.08 [4.21–6.08]	54.14 [44.67–64.75]	-45.35% [-54.39% to -34.74%]
**Mississippi**	0.64 [0.53–0.77]	13.90 [11.60–16.62]	-15.24% [-30.90% to 3.47%]	0.08 [0.06–0.11]	1.76 [1.20–2.37]	-22.27% [-38.14% to -3.76%]	4.36 [3.68–5.20]	97.12 [82.07–115.80]	-30.85% [-42.22% to -16.71%]
**Missouri**	1.26 [1.06–1.50]	12.26 [10.32–14.51]	-15.67% [-30.53% to 2.97%]	0.15 [0.10–0.20]	1.45 [0.99–1.93]	-22.60% [-38.65% to -4.42%]	6.39 [5.39–7.50]	63.94 [53.92–75.20]	-35.79% [-46.71% to -22.83%]
**Montana**	0.22 [0.18–0.26]	11.06 [9.38–13.25]	-24.72% [-38.21% to -8.47%]	0.03 [0.02–0.04]	1.31 [0.92–1.76]	-30.39% [-43.65% to -13.01%]	1.07 [0.90–1.27]	56.43 [47.65–67.12]	-41.88% [-51.56% to -30.38%]
**Nebraska**	0.38 [0.31–0.45]	11.96 [9.87–14.16]	-23.92% [-36.93% to -7.77%]	0.04 [0.03–0.06]	1.36 [0.96–1.86]	-29.58% [-42.37% to -14.28%]	1.65 [1.37–1.95]	53.77 [44.50–63.46]	-42.72% [-51.96% to -31.68%]
**Nevada**	0.60 [0.49–0.70]	12.17 [10.11–14.30]	-25.36% [-38.87% to -9.83%]	0.07 [0.05–0.09]	1.42 [0.99–1.85]	-34.02% [-46.47% to -18.18%]	2.95 [2.46–3.44]	61.71 [51.59–72.20]	-49.10% [-57.66% to -40.63%]
**New Hampshire**	0.31 [0.26–0.38]	12.11 [10.01–14.63]	-32.52% [-44.78% to -18.43%]	0.04 [0.02–0.05]	1.36 [0.93–1.87]	-38.20% [-50.44% to -21.25%]	1.29 [1.07–1.55]	51.61 [43.18–61.83]	-51.15% [-58.99% to -41.50%]
**New Jersey**	2.56 [2.09–3.06]	16.63 [13.62–19.85]	-37.04% [-48.12% to -24.66%]	0.28 [0.20–0.38]	1.79 [1.26–2.42]	-43.22% [-54.03% to -28.21%]	9.98 [8.20–11.84]	65.14 [53.58–77.41]	-56.50% [-64.05% to -48.06%]
**New Mexico**	0.55 [0.46–0.65]	16.02 [13.43–18.81]	-10.36% [-25.24% to 7.97%]	0.07 [0.04–0.09]	1.86 [1.25–2.52]	-19.64% [-35.66% to -0.89%]	3.00 [2.53–3.53]	90.79 [76.21–106.42]	-32.71% [-43.84% to -20.60%]
**New York**	5.34 [4.44–6.27]	15.95 [13.27–18.67]	-17.49% [-31.79% to -2.87%]	0.60 [0.42–0.79]	1.75 [1.23–2.31]	-30.87% [-44.87% to -15.85%]	22.81 [19.02–26.67]	69.95 [58.85–81.63]	-51.97% [-59.76% to -44.06%]
**North Carolina**	2.17 [1.82–2.57]	12.63 [10.62–15.00]	-17.11% [-31.14% to -0.36%]	0.26 [0.18–0.35]	1.48 [1.03–2.02]	-27.00% [-41.17% to -11.07%]	11.02 [9.19–12.84]	66.30 [55.85–77.38]	-43.17% [-51.66% to -33.59%]

**North Dakota**	0.14 [0.12–0.17]	11.56 [9.53–13.69]	-37.63% [-48.19% to -24.93%]	0.02 [0.01–0.02]	1.34 [0.90–1.83]	-40.94% [-52.69% to -27.76%]	0.69 [0.57–0.81]	56.78 [47.56–66.27]	-49.11% [-57.08% to -39.50%]
**Ohio**	2.29 [1.91–2.67]	11.40 [9.53–13.35]	-31.34% [-42.46% to -18.61%]	0.28 [0.20–0.37]	1.36 [0.98–1.83]	-36.09% [-47.18% to -23.05%]	11.95 [10.09–13.88]	61.54 [51.93–71.52]	-45.57% [-53.94% to -36.85%]
**Oklahoma**	0.68 [0.57–0.81]	11.00 [9.29–13.15]	-29.15% [-41.75% to -14.08%]	0.09 [0.06–0.12]	1.37 [0.95–1.93]	-31.20% [-45.34% to -15.26%]	4.02 [3.44–4.69]	67.23 [57.65–78.40]	-37.17% [-47.06% to -25.14%]
**Oregon**	0.92 [0.76–1.10]	12.12 [10.05–14.55]	-24.05% [-37.86% to -9.66%]	0.10 [0.07–0.15]	1.35 [0.92–1.92]	-30.76% [-45.16% to -15.35%]	3.76 [3.13–4.50]	51.72 [43.02–61.97]	-45.11% [-54.45% to -35.03%]
**Pennsylvania**	3.11 [2.56–3.71]	13.37 [11.11–15.83]	-32.78% [-44.55% to -19.55%]	0.37 [0.25–0.51]	1.54 [1.06–2.15]	-38.59% [-49.68% to -24.06%]	14.43 [12.06–16.95]	63.98 [53.18–75.00]	-51.95% [-60.14% to -43.34%]
**Rhode Island**	0.29 [0.24–0.35]	14.87 [12.33–17.88]	-35.21% [-46.84% to -22.37%]	0.03 [0.02–0.05]	1.65 [1.13–2.29]	-41.86% [-53.49% to -27.26%]	1.24 [1.04–1.48]	64.36 [54.22–76.85]	-56.18% [-63.22% to -48.37%]
**South Carolina**	1.20 [0.98–1.42]	13.95 [11.49–16.47]	-12.16% [-27.14% to 4.68%]	0.15 [0.10–0.20]	1.67 [1.17–2.27]	-23.02% [-38.71% to -5.39%]	6.73 [5.61–7.88]	81.58 [67.88–96.05]	-40.57% [-50.01% to -29.60%]
**South Dakota**	0.16 [0.14–0.19]	11.11 [9.42–13.18]	-27.23% [-38.92% to -12.34%]	0.02 [0.01–0.03]	1.31 [0.93–1.79]	-32.21% [-45.18% to -17.05%]	0.82 [0.70–0.95]	58.62 [50.32–67.75]	-41.50% [-49.49% to -31.17%]
**Tennessee**	1.35 [1.14–1.58]	11.97 [10.12–14.01]	-16.96% [-30.65% to -0.95%]	0.17 [0.12–0.22]	1.46 [1.02–1.96]	-24.00% [-38.62% to -6.54%]	7.64 [6.49–8.95]	70.01 [59.57–82.13]	-36.64% [-46.41% to -24.95%]
**Texas**	6.12 [5.20–7.13]	14.85 [12.65–17.27]	-16.65% [-28.99% to -1.95%]	0.70 [0.49–0.97]	1.70 [1.19–2.33]	-25.72% [-39.20% to -10.33%]	30.14 [25.75–34.58]	74.60 [63.82–85.39]	-41.11% [-49.45% to -31.65%]
**Utah**	0.43 [0.36–0.50]	10.58 [8.87–12.43]	-20.53% [-32.92% to -5.68%]	0.05 [0.04–0.07]	1.24 [0.86–1.69]	-28.21% [-41.46% to -10.96%]	2.00 [1.69–2.31]	50.32 [42.54–58.12]	-43.37% [-51.88% to -33.88%]
**Vermont**	0.14 [0.12–0.16]	11.06 [9.52–13.05]	-23.86% [-35.48% to -9.53%]	0.02 [0.01–0.02]	1.26 [0.91–1.66]	-32.66% [-44.37% to -17.31%]	0.59 [0.51–0.68]	49.83 [43.52–57.99]	-49.41% [-56.30% to -41.21%]
**Virginia**	1.97 [1.65–2.34]	14.04 [11.87–16.76]	-19.04% [-32.98% to -2.92%]	0.22 [0.15–0.30]	1.57 [1.09–2.12]	-29.00% [-42.79% to -14.23%]	8.70 [7.32–10.21]	63.71 [53.86–74.59]	-45.61% [-54.77% to -36.06%]

**Washington**	1.57 [1.29–1.86]	12.43 [10.25–14.60]	-27.87% [-39.98% to -13.57%]	0.18 [0.12–0.24]	1.39 [0.96–1.85]	-35.47% [-47.21% to -21.33%]	6.38 [5.32–7.48]	51.29 [42.71–59.91]	-51.08% [-59.08% to -41.95%]
**West Virginia**	0.37 [0.32–0.44]	11.69 [9.94–13.79]	-20.44% [-33.40% to -4.97%]	0.05 [0.03–0.06]	1.45 [1.02–1.97]	-25.34% [-38.74% to -9.61%]	2.22 [1.88–2.64]	72.52 [61.37–85.75]	-33.57% [-44.14% to -20.89%]
**Wisconsin**	1.34 [1.10–1.64]	13.18 [10.79–16.09]	-32.42% [-45.05% to -17.42%]	0.15 [0.11–0.21]	1.48 [1.02–2.03]	-37.33% [-50.73% to -23.13%]	5.80 [4.81–6.99]	58.50 [48.57–70.44]	-46.89% [-56.30% to -36.69%]
**Wyoming**	0.11 [0.09–0.13]	11.36 [9.68–13.15]	-19.26% [-32.27% to -4.64%]	0.01 [0.01–0.02]	1.33 [0.91–1.76]	-26.95% [-41.01% to -11.92%]	0.52 [0.45–0.60]	56.71 [48.79–65.03]	-40.51% [-48.79% to -31.17%]

eTable 3a: Incidence and death rate of gastric cancer, by age and sex, United States, 2021

	Incidence (95% UI)				Deaths (95% UI)			
	Male		Female		Male		Female	
	**Rate, per 100,000 people, 2021**	**Percentage Change, 1990-2021**	**Rate, per 100,000 people, 2021**	**Percentage Change, 1990-2021**	**Rate, per 100,000 people, 2021**	**Percentage Change, 1990-2021**	**Rate, per 100,000 people, 2021**	**Percentage Change, 1990-2021**
Age-standardized	6.79 [6.37–7.07]	-42.01% [-44.28% to -39.75%]	3.60 [3.27–3.80]	-32.22% [-34.51% to -29.72%]	3.76 [3.50–3.93]	-51.59% [-53.53% to -49.67%]	2.07 [1.86–2.20]	-42.97% [-44.76% to -40.98%]
15-19 years	0.06 [0.05–0.06]	-41.88% [-46.24% to -35.76%]	0.05 [0.04–0.05]	-46.44% [-50.07% to -42.63%]	0.01 [0.01–0.02]	-55.14% [-57.75% to -49.90%]	0.01 [0.01–0.02]	-55.45% [-57.44% to -53.42%]
20-24 years	0.24 [0.22–0.25]	-7.98% [-15.60% to -0.67%]	0.21 [0.19–0.22]	-6.46% [-13.89% to 1.82%]	0.07 [0.06–0.07]	-29.23% [-34.27% to -25.24%]	0.07 [0.07–0.08]	-22.04% [-26.55% to -16.73%]
25-29 years	0.81 [0.75–0.85]	12.50% [3.27% to 21.41%]	0.62 [0.57–0.66]	-7.64% [-15.73% to 0.09%]	0.22 [0.21–0.23]	-8.20% [-15.21% to -2.39%]	0.20 [0.19–0.21]	-21.30% [-26.66% to -15.75%]
30-34 years	1.44 [1.36–1.51]	-5.44% [-12.18% to 0.57%]	1.19 [1.11–1.27]	3.60% [-5.17% to 12.04%]	0.44 [0.42–0.46]	-23.52% [-28.37% to -19.58%]	0.43 [0.41–0.46]	-11.48% [-17.35% to -5.13%]
35-39 years	2.40 [2.26–2.55]	-13.66% [-19.96% to -7.44%]	1.75 [1.65–1.87]	9.56% [1.24% to 18.70%]	0.92 [0.87–0.96]	-28.56% [-33.03% to -24.35%]	0.78 [0.74–0.82]	-4.47% [-10.81% to 2.83%]
40-44 years	3.72 [3.53–3.92]	-25.49% [-30.59% to -20.57%]	2.63 [2.49–2.78]	12.96% [5.21% to 20.85%]	1.59 [1.53–1.65]	-38.39% [-42.08% to -34.96%]	1.26 [1.20–1.32]	-1.36% [-7.10% to 4.74%]
45-49 years	6.07 [5.79–6.36]	-30.20% [-34.27% to -25.68%]	3.92 [3.71–4.15]	2.18% [-4.43% to 10.34%]	2.78 [2.66–2.89]	-42.21% [-45.33% to -39.19%]	1.96 [1.87–2.06]	-11.51% [-16.54% to -5.54%]
50-54 years	9.41 [9.01–9.86]	-38.19% [-42.05% to -34.53%]	5.08 [4.81–5.35]	-16.50% [-21.65% to -10.95%]	4.64 [4.44–4.81]	-48.51% [-51.32% to -45.97%]	2.65 [2.54–2.77]	-28.64% [-32.45% to -24.52%]
55-59 years	15.17 [14.42–15.92]	-40.29% [-43.68% to -36.85%]	7.31 [6.94–7.72]	-24.50% [-28.76% to -19.81%]	7.38 [7.07–7.69]	-50.71% [-53.03% to -48.09%]	3.69 [3.54–3.85]	-36.78% [-39.82% to -33.18%]
60-64 years	21.24 [20.20–22.31]	-42.55% [-46.10% to -38.88%]	9.19 [8.61–9.75]	-36.82% [-40.80% to -32.13%]	10.81 [10.32–11.25]	-52.22% [-54.87% to -49.76%]	4.81 [4.54–5.07]	-46.87% [-49.94% to -43.97%]

65-69 years	28.90 [27.20–30.42]	-46.23% [-49.52% to -42.82%]	12.63 [11.69–13.46]	-40.03% [-43.62% to -35.95%]	14.90 [14.11–15.56]	-55.22% [-57.47% to -52.85%]	6.65 [6.19–7.01]	-49.64% [-52.31% to -46.79%]
70-74 years	38.88 [36.17–41.13]	-48.24% [-51.38% to -44.73%]	17.58 [15.76–18.84]	-43.47% [-47.15% to -39.84%]	20.78 [19.59–21.84]	-56.53% [-58.78% to -53.92%]	9.57 [8.62–10.17]	-52.15% [-54.95% to -49.17%]
75-79 years	49.72 [45.59–52.90]	-47.57% [-51.06% to -44.02%]	26.28 [22.70–28.42]	-41.94% [-45.67% to -37.64%]	29.35 [27.12–31.03]	-55.46% [-58.21% to -52.37%]	15.85 [13.90–17.07]	-50.22% [-52.83% to -47.11%]
80-84 years	61.39 [53.03–66.30]	-44.66% [-49.01% to -40.60%]	34.44 [27.29–38.49]	-39.49% [-43.79% to -35.02%]	41.47 [36.17–44.56]	-52.27% [-55.51% to -49.00%]	23.79 [19.09–26.50]	-47.42% [-50.80% to -44.14%]
85-89 years	79.83 [65.40–88.02]	-41.01% [-44.92% to -37.52%]	47.11 [35.27–53.79]	-38.54% [-43.41% to -33.92%]	58.04 [47.63–63.47]	-49.10% [-52.44% to -46.41%]	34.81 [25.97–39.61]	-46.24% [-49.92% to -42.49%]
90-94 years	85.81 [67.85–95.79]	-41.80% [-45.39% to -39.01%]	56.13 [40.70–65.24]	-36.90% [-41.21% to -33.72%]	74.91 [59.27–83.49]	-48.94% [-51.73% to -46.91%]	49.34 [35.68–57.11]	-44.07% [-47.11% to -41.70%]
95+ years	77.12 [56.19–88.45]	-38.88% [-43.39% to -36.53%]	58.05 [40.13–67.58]	-32.80% [-35.79% to -30.22%]	84.55 [62.02–97.19]	-44.15% [-48.28% to -42.14%]	64.00 [44.21–74.55]	-38.05% [-40.55% to -35.88%]

eTable 3b: DALY and prevalence rate of gastric cancer, by age and sex, United States, 2021

	DALYs (95% UI)				Prevalence (95% UI)			
	Male		Female		Male		Female	
	**Rate, per 100,000 people, 2021**	**Percentage Change, 1990-2021**	**Rate, per 100,000 people, 2021**	**Percentage Change, 1990-2021**	**Rate, per 100,000 people, 2021**	**Percentage Change, 1990-2021**	**Rate, per 100,000 people, 2021**	**Percentage Change, 1990-2021**
Age-standardized	89.92 [85.76–93.21]	-49.84% [-51.81% to -47.93%]	50.90 [47.65–53.33]	-37.68% [-39.66% to -35.56%]	18.79 [17.80–19.56]	-28.25% [-31.42% to -25.23%]	9.57 [8.87–10.04]	-16.02% [-18.94% to -12.99%]
15-19 years	1.10 [1.05–1.18]	-54.92% [-57.53% to -49.67%]	1.10 [1.05–1.14]	-55.32% [-57.31% to -53.29%]	0.20 [0.19–0.21]	-35.17% [-40.36% to -27.62%]	0.16 [0.15–0.17]	-38.75% [-43.36% to -34.29%]
20-24 years	4.64 [4.40–4.86]	-28.95% [-33.99% to -24.99%]	4.91 [4.66–5.16]	-21.89% [-26.35% to -16.57%]	0.86 [0.80–0.92]	2.89% [-6.41% to 12.03%]	0.72 [0.66–0.77]	6.66% [-2.70% to 16.20%]
25-29 years	14.11 [13.16–14.83]	-7.70% [-14.67% to -1.83%]	12.69 [11.91–13.50]	-21.00% [-26.41% to -15.39%]	3.47 [3.22–3.68]	22.71% [12.17% to 32.75%]	2.45 [2.28–2.62]	2.31% [-7.10% to 10.72%]
30-34 years	26.02 [24.83–27.08]	-23.06% [-27.97% to -19.11%]	25.53 [24.10–27.05]	-11.17% [-17.01% to -4.76%]	5.71 [5.36–6.04]	2.77% [-4.70% to 10.41%]	4.39 [4.07–4.70]	14.40% [4.26% to 23.92%]
35-39 years	49.22 [46.73–51.67]	-28.32% [-32.83% to -24.09%]	41.67 [39.33–44.27]	-4.24% [-10.60% to 3.05%]	8.56 [8.05–9.07]	-3.77% [-11.57% to 3.80%]	6.06 [5.68–6.47]	24.29% [14.15% to 35.17%]
40-44 years	77.49 [74.35–80.63]	-38.18% [-41.86% to -34.69%]	61.25 [58.28–64.49]	-1.09% [-6.86% to 4.97%]	13.56 [12.76–14.38]	-12.61% [-19.43% to -5.41%]	9.02 [8.49–9.61]	32.54% [22.55% to 42.56%]
45-49 years	121.35 [116.11–126.08]	-42.07% [-45.21% to -39.14%]	85.57 [81.45–89.86]	-11.36% [-16.37% to -5.40%]	20.16 [19.07–21.46]	-17.71% [-23.78% to -11.12%]	12.83 [12.01–13.60]	20.22% [11.87% to 30.39%]
50-54 years	180.34 [173.06–187.39]	-48.27% [-51.11% to -45.73%]	102.93 [98.49–107.66]	-28.36% [-32.10% to -24.25%]	30.22 [28.54–32.20]	-26.73% [-32.16% to -21.04%]	15.58 [14.68–16.57]	-1.06% [-8.25% to 6.58%]
55-59 years	252.06 [241.38–263.32]	-50.43% [-52.72% to -47.80%]	125.69 [120.65–131.24]	-36.46% [-39.50% to -32.80%]	47.85 [45.26–50.73]	-28.78% [-33.82% to -23.51%]	22.48 [21.21–23.88]	-9.43% [-15.56% to -3.15%]

60-64 years	319.55 [305.41–333.14]	-51.83% [-54.54% to -49.37%]	141.94 [133.50–149.38]	-46.48% [-49.56% to -43.55%]	65.36 [61.44–69.52]	-30.58% [-35.90% to -24.16%]	27.78 [25.93–29.64]	-23.77% [-29.45% to -17.34%]
65-69 years	372.49 [353.04–389.34]	-54.77% [-57.00% to -52.36%]	165.55 [154.33–174.58]	-49.27% [-51.99% to -46.44%]	86.53 [80.47–91.52]	-33.92% [-38.98% to -28.34%]	35.93 [32.92–38.43]	-27.35% [-32.41% to -21.87%]
70-74 years	426.37 [400.18–448.80]	-56.32% [-58.56% to -53.69%]	196.10 [177.01–208.94]	-51.93% [-54.74% to -48.98%]	108.02 [99.98–115.20]	-35.72% [-41.17% to -30.80%]	47.32 [42.19–51.05]	-30.75% [-35.99% to -25.73%]
75-79 years	481.99 [446.38–509.92]	-55.28% [-58.16% to -52.19%]	260.11 [228.30–279.83]	-49.83% [-52.53% to -46.66%]	123.41 [112.66–133.39]	-33.53% [-39.03% to -27.43%]	62.64 [53.95–68.11]	-28.80% [-34.55% to -22.56%]
80-84 years	533.74 [465.72–574.44]	-52.12% [-55.42% to -48.81%]	305.64 [246.39–341.61]	-47.02% [-50.38% to -43.58%]	136.16 [117.69–148.19]	-28.34% [-35.57% to -21.16%]	73.26 [58.26–83.32]	-25.44% [-31.55% to -18.19%]
85-89 years	592.07 [485.70–646.65]	-48.97% [-52.29% to -46.20%]	353.72 [264.86–403.63]	-46.05% [-49.73% to -42.24%]	123.44 [100.82–137.23]	-23.93% [-31.07% to -17.07%]	69.26 [51.69–80.55]	-25.95% [-33.58% to -18.16%]
90-94 years	663.34 [523.73–739.48]	-48.69% [-51.48% to -46.56%]	436.57 [316.18–505.66]	-43.84% [-46.86% to -41.35%]	102.69 [81.08–115.13]	-29.10% [-35.01% to -24.37%]	63.78 [45.80–74.72]	-25.83% [-31.80% to -20.24%]
95+ years	703.24 [515.30–808.58]	-43.83% [-48.04% to -41.85%]	529.53 [366.02–615.82]	-37.71% [-40.17% to -35.54%]	42.78 [31.13–49.06]	-36.51% [-41.04% to -33.73%]	31.33 [21.65–36.61]	-30.54% [-33.69% to -27.78%]

eTable 3c: YLD and YLL rate of gastric cancer, by age and sex, United States, 2021

	YLDs (95% UI)				YLLs (95% UI)			
	Male		Female		Male		Female	
	**Rate, per 100,000 people, 2021**	**Percentage Change, 1990-2021**	**Rate, per 100,000 people, 2021**	**Percentage Change, 1990-2021**	**Rate, per 100,000 people, 2021**	**Percentage Change, 1990-2021**	**Rate, per 100,000 people, 2021**	**Percentage Change, 1990-2021**
Age-standardized	2.12 [1.57–2.73]	-35.62% [-39.47% to -31.47%]	1.12 [0.82–1.47]	-25.10% [-28.51% to -20.78%]	87.80 [83.62–91.07]	-50.10% [-52.07% to -48.22%]	49.79 [46.47–52.05]	-37.92% [-39.89% to -35.81%]
15-19 years	0.02 [0.02–0.03]	-38.87% [-43.41% to -31.94%]	0.02 [0.01–0.02]	-43.30% [-47.14% to -39.15%]	1.08 [1.03–1.16]	-55.14% [-57.75% to -49.89%]	1.08 [1.04–1.13]	-55.46% [-57.45% to -53.44%]
20-24 years	0.09 [0.07–0.12]	-3.13% [-10.89% to 5.22%]	0.08 [0.05–0.10]	-0.77% [-8.81% to 8.43%]	4.55 [4.32–4.77]	-29.32% [-34.36% to -25.34%]	4.84 [4.59–5.08]	-22.15% [-26.65% to -16.85%]
25-29 years	0.34 [0.24–0.45]	17.80% [8.11% to 27.49%]	0.24 [0.17–0.32]	-2.89% [-11.94% to 5.03%]	13.77 [12.88–14.49]	-8.19% [-15.19% to -2.38%]	12.45 [11.69–13.23]	-21.28% [-26.64% to -15.74%]
30-34 years	0.58 [0.42–0.77]	-1.30% [-8.19% to 5.56%]	0.44 [0.32–0.58]	8.43% [-1.09% to 17.37%]	25.44 [24.24–26.46]	-23.45% [-28.31% to -19.51%]	25.09 [23.68–26.58]	-11.45% [-17.31% to -5.09%]
35-39 years	0.89 [0.63–1.19]	-9.16% [-16.98% to -2.28%]	0.63 [0.46–0.84]	15.91% [6.75% to 25.82%]	48.33 [45.88–50.64]	-28.60% [-33.07% to -24.39%]	41.04 [38.80–43.51]	-4.50% [-10.84% to 2.80%]
40-44 years	1.36 [0.98–1.81]	-19.92% [-30.45% to -6.56%]	0.93 [0.67–1.22]	20.22% [10.51% to 31.76%]	76.13 [73.04–79.18]	-38.43% [-42.12% to -35.00%]	60.32 [57.39–63.41]	-1.36% [-7.11% to 4.73%]
45-49 years	2.13 [1.55–2.89]	-25.19% [-33.92% to -15.21%]	1.36 [0.99–1.79]	8.18% [-3.65% to 22.89%]	119.22 [114.18–124.03]	-42.30% [-45.43% to -39.30%]	84.21 [80.15–88.54]	-11.62% [-16.63% to -5.65%]
50-54 years	3.29 [2.38–4.44]	-33.69% [-41.61% to -26.11%]	1.69 [1.22–2.27]	-11.38% [-23.48% to 0.93%]	177.05 [169.71–183.86]	-48.48% [-51.29% to -45.94%]	101.24 [96.95–105.93]	-28.59% [-32.39% to -24.45%]
55-59 years	5.25 [3.78–6.75]	-35.91% [-41.81% to -29.70%]	2.41 [1.74–3.20]	-19.67% [-28.80% to -7.40%]	246.82 [236.52–257.26]	-50.67% [-52.98% to -48.04%]	123.28 [118.25–128.68]	-36.72% [-39.77% to -33.12%]

60-64 years	7.24 [5.26–9.61]	-36.57% [-43.35% to -28.71%]	3.05 [2.24–3.99]	-32.27% [-40.15% to -24.39%]	312.31 [298.03–324.88]	-52.10% [-54.76% to -49.63%]	138.89 [130.93–146.23]	-46.73% [-49.81% to -43.82%]
65-69 years	9.70 [7.08–12.79]	-36.80% [-45.11% to -27.23%]	4.04 [2.92–5.32]	-36.15% [-42.48% to -29.84%]	362.79 [343.45–378.76]	-55.11% [-57.36% to -52.73%]	161.52 [150.38–170.26]	-49.53% [-52.21% to -46.68%]
70-74 years	11.89 [8.50–15.55]	-42.20% [-51.36% to -31.11%]	5.55 [4.00–7.41]	-38.73% [-45.03% to -31.95%]	414.48 [390.68–435.59]	-56.62% [-58.87% to -54.02%]	190.55 [171.69–202.61]	-52.23% [-55.02% to -49.25%]
75-79 years	13.88 [10.21–17.86]	-43.53% [-52.80% to -33.11%]	7.97 [5.61–10.65]	-31.79% [-40.90% to -21.91%]	468.10 [432.50–494.94]	-55.55% [-58.30% to -52.48%]	252.14 [221.14–271.60]	-50.25% [-52.85% to -47.13%]
80-84 years	16.28 [11.89–21.37]	-39.75% [-49.20% to -29.02%]	9.68 [6.66–12.92]	-30.48% [-40.03% to -18.54%]	517.45 [451.31–556.03]	-52.43% [-55.66% to -49.17%]	295.96 [237.54–329.66]	-47.43% [-50.81% to -44.16%]
85-89 years	18.32 [12.61–23.89]	-35.08% [-44.85% to -23.08%]	10.92 [7.29–14.99]	-34.26% [-46.21% to -22.57%]	573.75 [470.85–627.44]	-49.31% [-52.63% to -46.64%]	342.79 [255.73–390.03]	-46.36% [-50.02% to -42.61%]
90-94 years	18.20 [12.51–24.04]	-36.24% [-46.31% to -24.74%]	12.00 [7.59–16.59]	-32.75% [-45.97% to -18.72%]	645.13 [510.39–719.00]	-48.97% [-51.75% to -46.94%]	424.57 [307.02–491.42]	-44.10% [-47.14% to -41.74%]
95+ years	13.94 [9.12–19.33]	-34.54% [-45.26% to -23.35%]	10.54 [6.26–15.13]	-32.32% [-44.88% to -19.75%]	689.30 [505.71–792.32]	-43.99% [-48.14% to -41.99%]	518.99 [358.54–604.34]	-37.81% [-40.32% to -35.64%]

eTable 4: Proportion of risk-attributable DALYs for gastric cancer, by age and sex, United States, 2021

	Smoking				Diet high in sodium			
	Male		Female		Male		Female	
	Attributable percentage, 2021	Percentage Change, 1990-2021	Attributable percentage, 2021	Percentage Change, 1990-2021	Attributable percentage, 2021	Percentage Change, 1990-2021	Attributable percentage, 2021	Percentage Change, 1990-2021
**30-34 years**	7.13% [5.73% to 8.85%]	-31.42% [-41.80 to -18.69]	5.13% [3.77% to 6.86%]	-41.52% [-53.97 to -25.51]	8.25% [0.00% to 41.33%]	9.09% [-0.95 to 9942.34]	7.86% [0.00% to 39.97%]	0.34% [-122.88 to 597.91]
**35-39 years**	8.13% [6.45% to 9.98%]	-34.29% [-43.12 to -24.95]	6.19% [4.60% to 8.06%]	-35.20% [-49.01 to -17.84]	8.20% [0.00% to 41.16%]	7.79% [-978.51 to 4934.82]	7.80% [0.00% to 40.10%]	-0.01% [-130.58 to 537.11]
**40-44 years**	8.78% [6.90% to 10.76%]	-37.14% [-46.24 to -27.56]	6.44% [4.79% to 8.40%]	-39.62% [-52.63 to -23.88]	8.22% [0.00% to 41.37%]	10.46% [-814.76 to 9847.97]	7.79% [0.00% to 39.82%]	1.41% [-200.82 to 966.14]
**45-49 years**	9.31% [7.43% to 11.30%]	-42.35% [-51.29 to -33.19]	6.93% [5.29% to 9.05%]	-43.43% [-55.79 to -27.78]	8.28% [0.00% to 41.45%]	12.45% [-0.23 to 12768.60]	7.72% [0.00% to 39.45%]	2.02% [-87.58 to 722.39]
**50-54 years**	11.13% [8.98% to 13.63%]	-43.26% [-52.74 to -33.56]	8.30% [6.29% to 10.77%]	-36.10% [-50.81 to -20.72]	8.18% [0.00% to 41.00%]	12.11% [-1410.69 to 12330.11]	7.68% [0.00% to 39.38%]	0.68% [-95.66 to 580.45]
**55-59 years**	13.63% [10.88% to 16.59%]	-39.39% [-49.57 to -27.32]	9.70% [7.42% to 12.21%]	-33.78% [-47.90 to -16.52]	8.18% [0.00% to 41.35%]	14.90% [-204.43 to 26512.82]	7.60% [0.00% to 39.70%]	1.05% [-103.36 to 831.68]
**60-64 years**	16.01% [12.52% to 19.79%]	-35.54% [-45.77 to -23.91]	10.09% [7.37% to 13.03%]	-34.75% [-48.20 to -15.70]	8.16% [0.00% to 41.19%]	17.01% [-788.67 to 20841.93]	7.49% [0.00% to 38.68%]	1.63% [-107.40 to 1271.72]
**65-69 years**	17.48% [13.69% to 21.59%]	-31.07% [-41.08 to -20.22]	10.71% [7.82% to 14.43%]	-33.31% [-48.97 to -12.71]	8.10% [0.00% to 40.89%]	7.63% [-116.35 to 2592.60]	7.42% [0.00% to 39.56%]	1.96% [-124.57 to 1630.16]
**70-74 years**	18.03% [13.92% to 22.99%]	-26.11% [-36.23 to -13.96]	11.89% [8.55% to 15.82%]	-22.05% [-39.75 to 1.27]	8.02% [0.00% to 40.56%]	16.91% [-343.59 to 20015.68]	7.26% [0.00% to 38.18%]	1.35% [-95.64 to 1383.85]
**75-79 years**	16.64% [12.37% to 21.51%]	-22.60% [-35.15 to -10.58]	11.56% [8.19% to 15.77%]	-8.70% [-32.50 to 18.98]	7.99% [0.00% to 40.70%]	20.57% [-253.77 to 58431.31]	7.07% [0.00% to 37.72%]	1.22% [-102.47 to 2186.94]

**80-84 years**	14.01% [10.24% to 18.85%]	-18.27% [-30.98 to -2.51]	8.97% [6.25% to 12.18%]	9.04% [-20.90 to 46.37]	7.83% [0.00% to 39.52%]	23.78% [-636.59 to 177325.69]	6.89% [0.00% to 36.25%]	1.09% [-97.75 to 1917.99]
**85-89 years**	12.32% [8.82% to 17.13%]	-20.80% [-34.72 to -4.30]	8.35% [5.58% to 11.85%]	4.58% [-26.11 to 43.65]	7.35% [0.00% to 39.17%]	-3.80% [-162.46 to 189.86]	6.64% [0.00% to 35.88%]	0.53% [-431.72 to 2281.70]
**90-94 years**	10.63% [7.40% to 15.20%]	-24.20% [-40.18 to -7.03]	7.63% [4.91% to 11.17%]	-0.93% [-32.19 to 40.77]	7.50% [0.00% to 39.34%]	2.45% [-98.05 to 1529.68]	6.61% [0.00% to 36.78%]	0.12% [-100.03 to 1914.66]
**95+ years**	7.18% [4.62% to 11.77%]	-34.56% [-52.08 to -14.30]	5.77% [3.24% to 9.05%]	-17.74% [-51.68 to 27.60]	7.52% [0.00% to 39.41%]	2.36% [-98.95 to 1807.18]	6.66% [0.00% to 37.61%]	0.54% [-98.82 to 3665.03]

eTable 5a: Results of secondary statistical analysis, multiple linear regression

Measure	Hispanic		White		Black		Asian		Aggregate Income		Educational Attainment	
	β (95% CI)	p-value	β (95% CI)	p-value	β (95% CI)	p-value	β (95% CI)	p-value	β (95% CI)	p-value	β (95% CI)	p-value
**Incidence**	0.063 [0.040 to 0.085]	<0.001	0.094 [0.064 to 0.124]	<0.001	0.111 [0.078 to 0.143]	<0.001	0.288 [0.179 to 0.396]	<0.001	-2.473 [-4.449 to -0.496]	0.015	0.344 [0.115 to 0.573]	0.004
**Deaths**	0.045 [0.030 to 0.060]	<0.001	0.068 [0.048 to 0.089]	<0.001	0.068 [0.046 to 0.090]	<0.001	0.200 [0.127 to 0.274]	<0.001	-1.747 [-3.082 to -0.413]	0.011	0.197 [0.042 to 0.351]	0.014
**DALYs**	1.293 [1.003 to 1.584]	<0.001	1.542 [1.150 to 1.934]	<0.001	1.901 [1.479 to 2.323]	<0.001	4.616 [3.200 to 6.032]	<0.001	-34.419 [-60.265 to -8.572]	0.010	3.097 [0.106 to 6.087]	0.043
**Prevalence**	0.138 [0.076 to 0.200]	<0.001	0.191 [0.107 to 0.275]	<0.001	0.233 [0.142 to 0.323]	<0.001	0.656 [0.354 to 0.959]	<0.001	-6.044 [-11.566 to -0.523]	0.033	1.050 [0.411 to 1.689]	0.002
**YLDs**	0.019 [0.012 to 0.026]	<0.001	0.027 [0.018 to 0.036]	<0.001	0.031 [0.021 to 0.041]	<0.001	0.081 [0.048 to 0.113]	<0.001	-0.742 [-1.340 to -0.144]	0.016	0.111 [0.042 to 0.180]	0.002
**YLLs**	1.274 [0.990 to 1.559]	<0.001	1.515 [1.130 to 1.900]	<0.001	1.870 [1.456 to 2.284]	<0.001	4.535 [3.146 to 5.924]	<0.001	-33.677 [-59.034 to -8.319]	0.010	2.986 [0.052 to 5.920]	0.046

eTable 5b: Results of secondary statistical analysis, mediation analysis, incidence

Variable	Hispanic		White		Black		Asian	
	β (95% CI)	p-value	β (95% CI)	p-value	β (95% CI)	p-value	β (95% CI)	p-value
Total Effect	0.053 [0.034 to 0.102]	0.008	0.084 [0.069 to 0.094]	<0.001	0.128 [0.097 to 0.177]	<0.001	0.220 [0.098 to 0.346]	<0.001
Direct Assocation	0.063 [0.030 to 0.125]	0.008	0.094 [0.048 to 0.126]	<0.001	0.111 [0.075 to 0.165]	<0.001	0.288 [0.030 to 0.448]	0.006
Indirect Assocation (Combined)	-0.009 [-0.036 to 0.020]	0.499	-0.010 [-0.044 to 0.030]	0.592	0.018 [-0.021 to 0.050]	0.334	-0.068 [-0.206 to 0.129]	0.430
Indirect Assocation (Income)	-0.058 [-0.109 to 0.033]	0.113	-0.098 [-0.180 to 0.046]	0.093	-0.072 [-0.168 to 0.024]	0.143	-0.293 [-0.694 to 0.178]	0.192
Indirect Assocation (Education)	0.048 [-0.029 to 0.090]	0.114	0.088 [-0.032 to 0.149]	0.066	0.090 [-0.023 to 0.191]	0.113	0.225 [-0.132 to 0.602]	0.209
Proportion Mediated (%)	12.500		9.615		13.953		19.101	

eTable 5c: Results of secondary statistical analysis, mediation analysis, deaths

Variable	Hispanic		White		Black		Asian	
	β (95% CI)	p-value	β (95% CI)	p-value	β (95% CI)	p-value	β (95% CI)	p-value
Total Effect	0.032 [0.021 to 0.064]	0.013	0.049 [0.039 to 0.056]	<0.001	0.068 [0.049 to 0.109]	<0.001	0.122 [-0.002 to 0.161]	0.004
Direct Assocation	0.045 [0.024 to 0.083]	0.003	0.068 [0.038 to 0.087]	<0.001	0.068 [0.043 to 0.111]	<0.001	0.200 [-0.046 to 0.279]	0.010
Indirect Assocation (Combined)	-0.013 [-0.030 to 0.007]	0.145	-0.019 [-0.039 to 0.012]	0.125	0.000 [-0.021 to 0.020]	0.970	-0.078 [-0.159 to 0.063]	0.151
Indirect Assocation (Income)	-0.041 [-0.075 to 0.017]	0.087	-0.069 [-0.124 to 0.025]	0.068	-0.051 [-0.106 to 0.013]	0.093	-0.207 [-0.434 to 0.119]	0.133
Indirect Assocation (Education)	0.028 [-0.020 to 0.055]	0.153	0.050 [-0.024 to 0.093]	0.098	0.051 [-0.019 to 0.107]	0.119	0.129 [-0.095 to 0.334]	0.229
Proportion Mediated (%)	22.414		21.839		0.000		28.058	

eTable 5d: Results of secondary statistical analysis, mediation analysis, prevalence

Variable	Hispanic		White		Black		Asian	
	β (95% CI)	p-value	β (95% CI)	p-value	β (95% CI)	p-value	β (95% CI)	p-value
Total Effect	0.145 [0.074 to 0.283]	0.014	0.219 [0.173 to 0.249]	<0.001	0.330 [0.219 to 0.455]	<0.001	0.628 [0.337 to 1.151]	0.002
Direct Assocation	0.138 [0.040 to 0.322]	0.039	0.191 [0.067 to 0.298]	0.001	0.233 [0.122 to 0.368]	<0.001	0.656 [0.076 to 1.323]	0.034
Indirect Assocation (Combined)	0.006 [-0.079 to 0.089]	0.886	0.028 [-0.092 to 0.144]	0.640	0.098 [-0.032 to 0.212]	0.123	-0.028 [-0.471 to 0.517]	0.910
Indirect Assocation (Income)	-0.141 [-0.296 to 0.110]	0.175	-0.240 [-0.475 to 0.163]	0.144	-0.176 [-0.457 to 0.084]	0.203	-0.715 [-1.963 to 0.571]	0.263
Indirect Assocation (Education)	0.147 [-0.069 to 0.277]	0.097	0.268 [-0.075 to 0.439]	0.047	0.274 [-0.052 to 0.609]	0.110	0.687 [-0.236 to 1.835]	0.181
Proportion Mediated (%)	4.167		12.785		29.607		4.094	

eTable 5e: Results of secondary statistical analysis, mediation analysis, YLDs

Variable	Hispanic		White		Black		Asian	
	β (95% CI)	p-value	β (95% CI)	p-value	β (95% CI)	p-value	β (95% CI)	p-value
Total Effect	0.017 [0.011 to 0.033]	0.007	0.026 [0.021 to 0.029]	<0.001	0.038 [0.029 to 0.052]	<0.001	0.065 [0.029 to 0.107]	<0.001
Direct Assocation	0.019 [0.009 to 0.039]	0.009	0.027 [0.013 to 0.037]	<0.001	0.031 [0.020 to 0.046]	<0.001	0.081 [0.008 to 0.131]	0.010
Indirect Assocation (Combined)	-0.002 [-0.010 to 0.007]	0.680	-0.001 [-0.012 to 0.012]	0.847	0.007 [-0.005 to 0.018]	0.222	-0.015 [-0.058 to 0.046]	0.556
Indirect Assocation (Income)	-0.017 [-0.034 to 0.010]	0.122	-0.030 [-0.054 to 0.014]	0.098	-0.022 [-0.050 to 0.007]	0.144	-0.088 [-0.206 to 0.051]	0.188
Indirect Assocation (Education)	0.016 [-0.007 to 0.029]	0.106	0.028 [-0.009 to 0.047]	0.055	0.029 [-0.006 to 0.060]	0.101	0.073 [-0.030 to 0.186]	0.183
Proportion Mediated (%)	9.524		3.571		18.421		15.625	

eTable 5f: Results of secondary statistical analysis, mediation analysis, YLLs

Variable	Hispanic		White		Black		Asian	
	β (95% CI)	p-value	β (95% CI)	p-value	β (95% CI)	p-value	β (95% CI)	p-value
Total Effect	0.908 [0.700 to 1.474]	<0.001	0.939 [0.758 to 1.074]	<0.001	1.667 [1.296 to 2.550]	<0.001	2.503 [-0.363 to 3.073]	0.005
Direct Assocation	1.274 [0.905 to 1.923]	<0.001	1.515 [0.951 to 1.863]	<0.001	1.870 [1.356 to 2.745]	<0.001	4.535 [-0.035 to 5.973]	0.001
Indirect Assocation (Combined)	-0.367 [-0.735 to 0.048]	0.048	-0.576 [-0.943 to 0.001]	0.013	-0.203 [-0.626 to 0.159]	0.293	-2.033 [-3.710 to 0.123]	0.036
Indirect Assocation (Income)	-0.785 [-1.447 to 0.313]	0.072	-1.339 [-2.338 to 0.403]	0.050	-0.981 [-2.130 to 0.209]	0.087	-3.986 [-8.228 to 1.895]	0.114
Indirect Assocation (Education)	0.418 [-0.447 to 0.877]	0.216	0.763 [-0.553 to 1.504]	0.148	0.778 [-0.444 to 1.751]	0.169	1.953 [-2.262 to 5.447]	0.303
Proportion Mediated (%)	22.364		27.547		9.793		30.953	

Appendix II: Supplementary figures

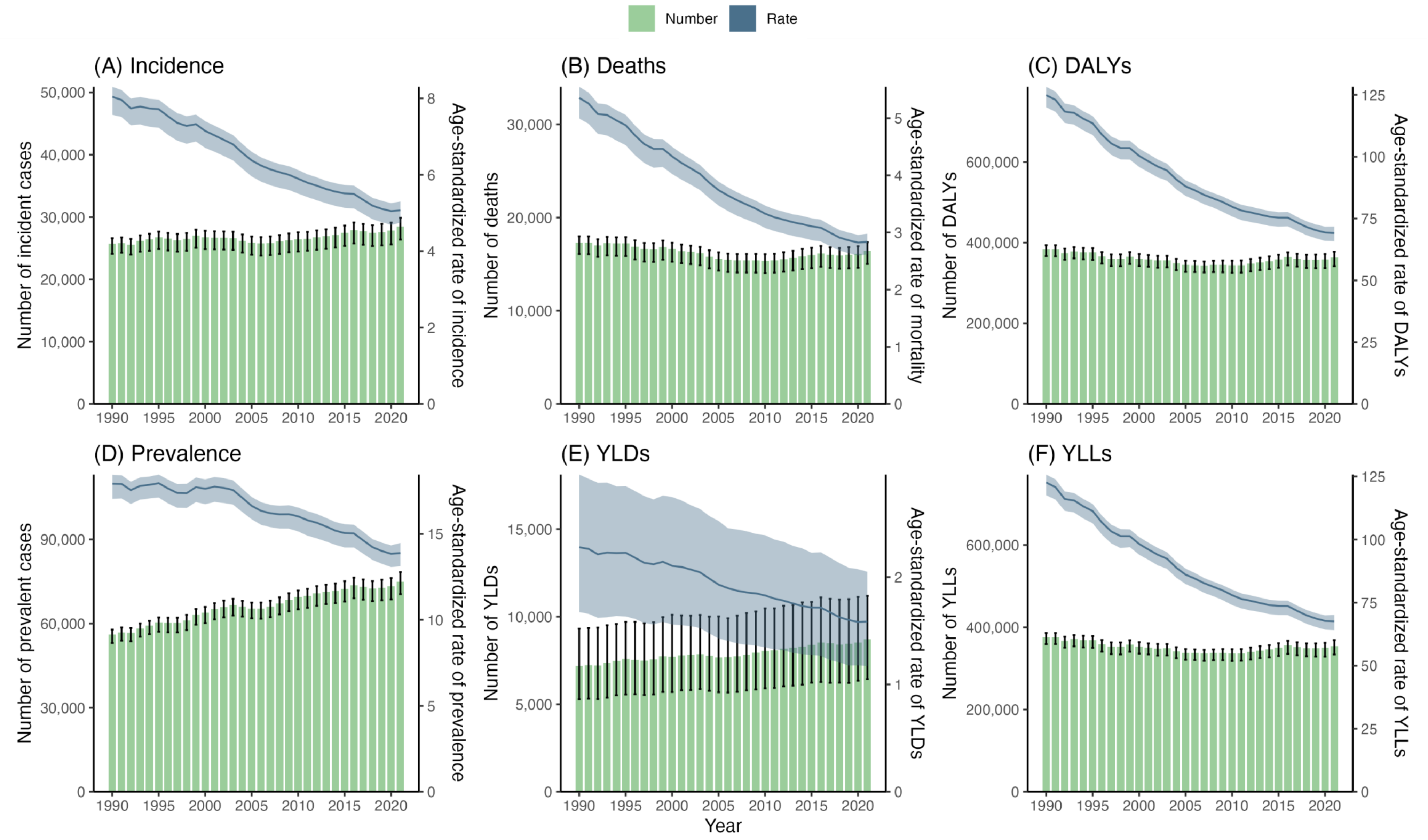

eFigure 1: Count and age-standardized rate of (A) incidence, (B) death, (C) DALY, (D) prevalence, (E) YLD, and (F) YLL of gastric cancer, 1990-2021, United States

(A) Prevalence

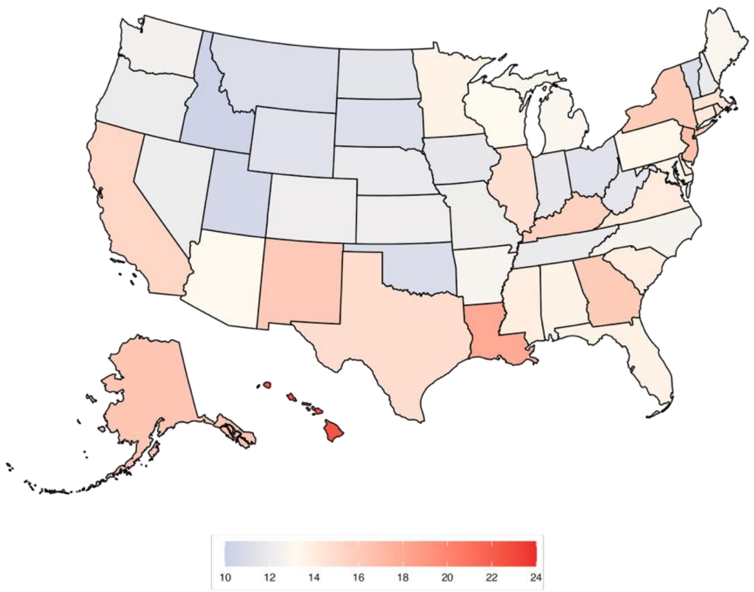

(B) YLDs

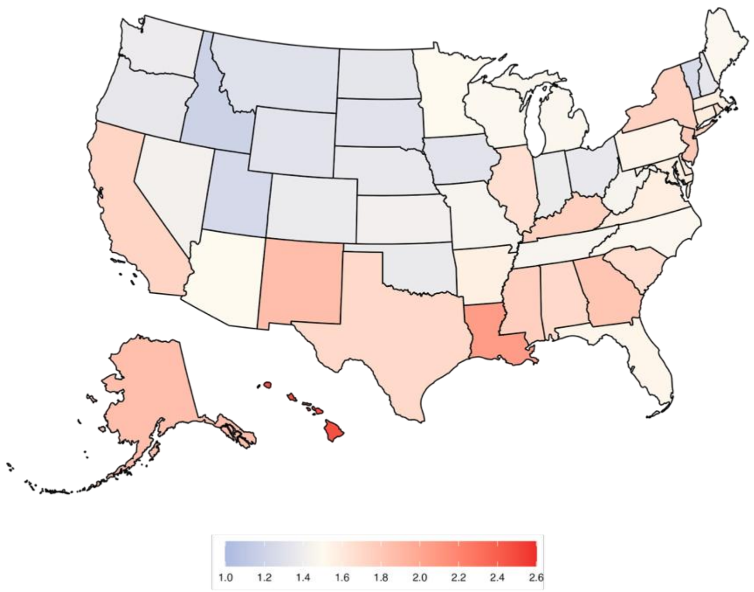

(C) YLLs

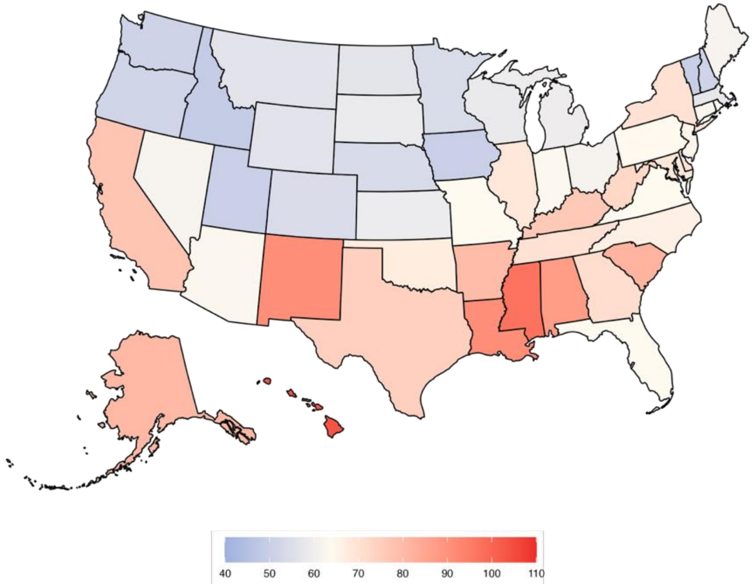

eFigure 2: Age-standardized rate of (A) prevalence, (B) YLDs, and (C) YLLs rate of gastric cancer, 2021, United States

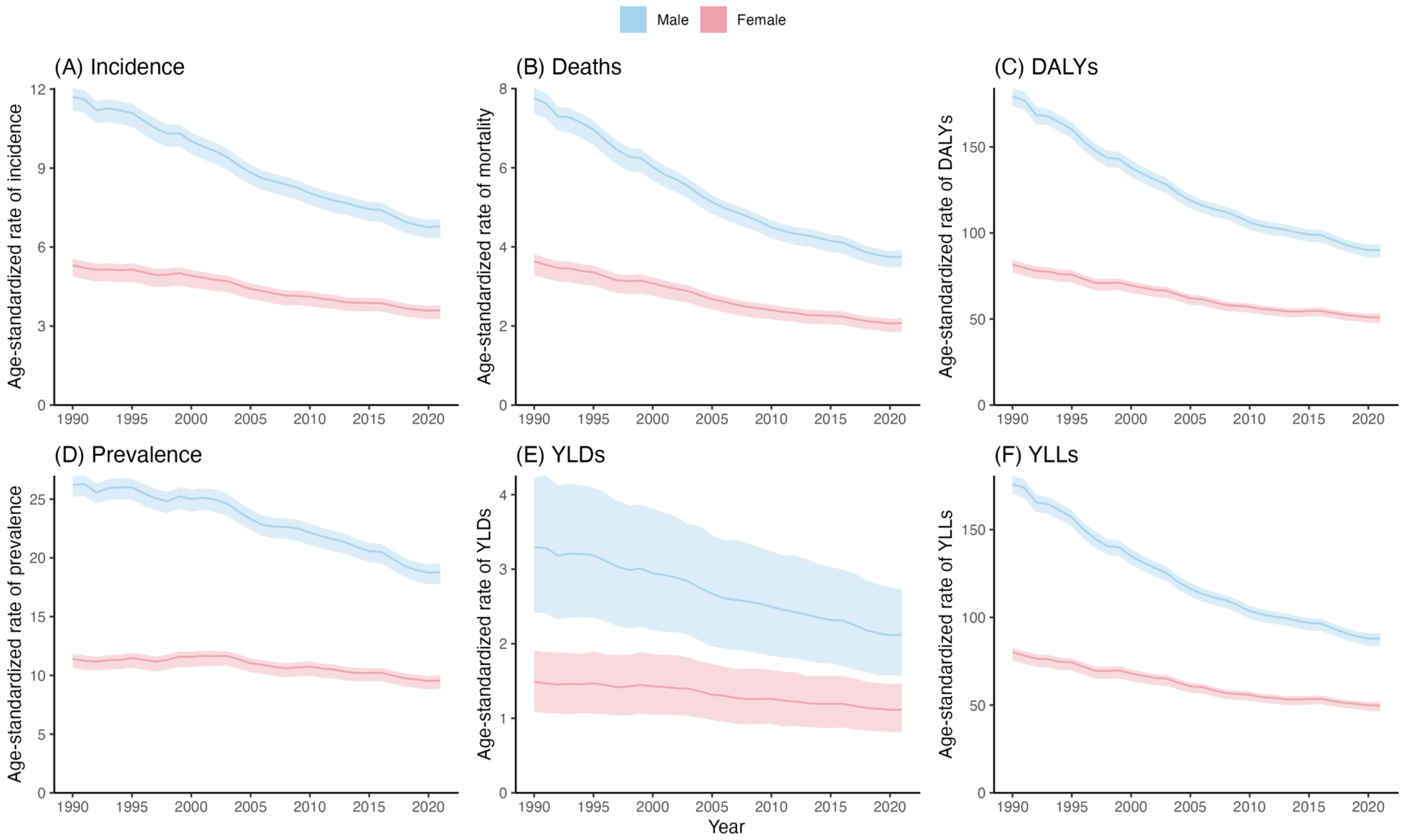

eFigure 3: Age-standardized rate of (A) incidence, (B) death, (C) DALY, (D) prevalence, (E) YLD, and (F) YLL of gastric cancer, male and female, 1990-2021, United States

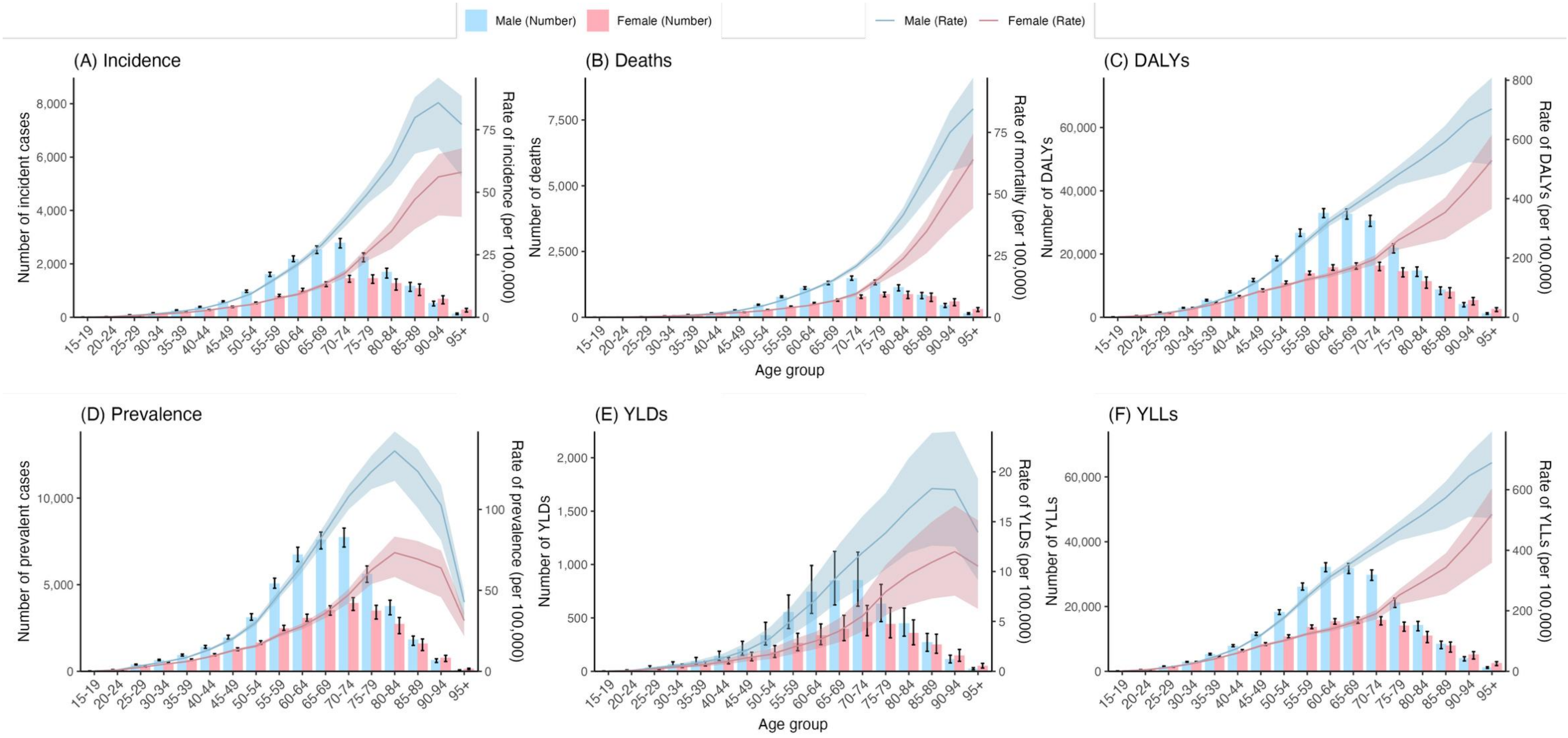

eFigure 4: Rate of (A) incidence, (B) death, (C) DALY, (D) prevalence, (E) YLD, and (F) YLL of gastric cancer by age-group, male and female, 2021, United States

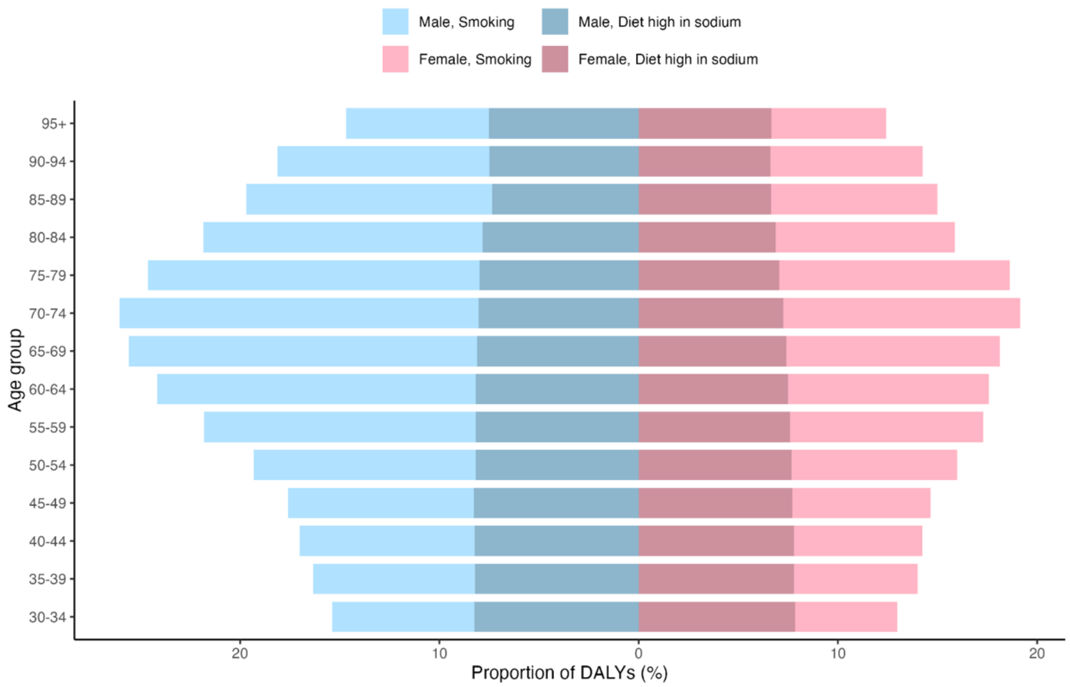

eFigure 5: Proportion of risk-attributable DALYs of Gastric Cancer, by age and sex, United States, 2021

(A) Incidence

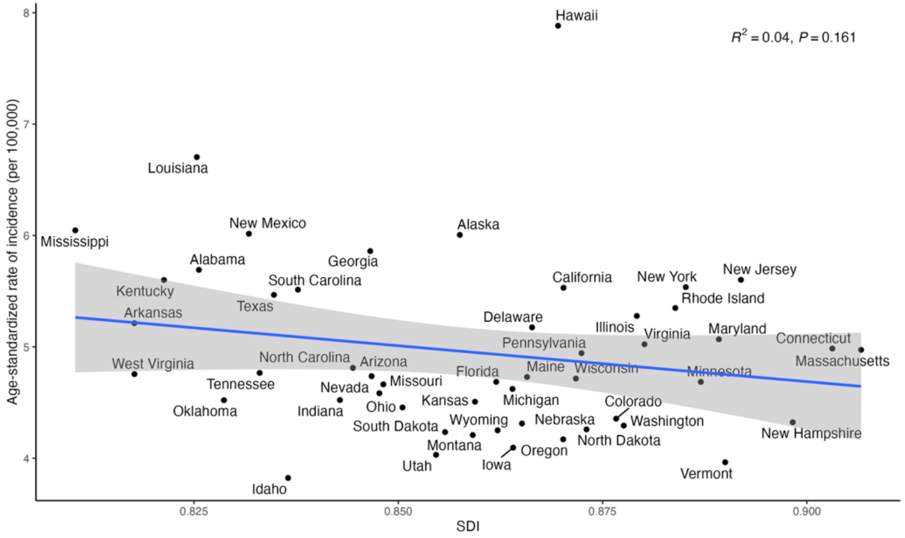

(B) Deaths

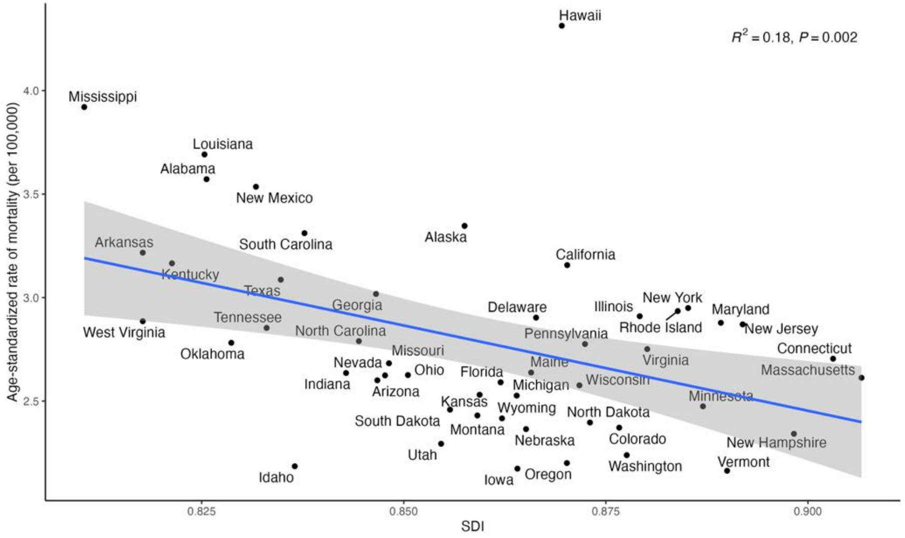

(C) Prevalence

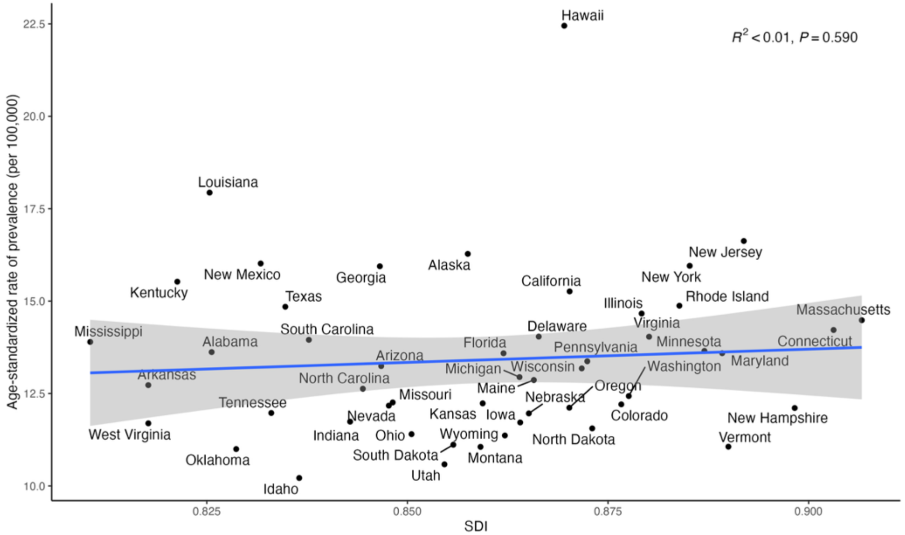

(D) YLDs

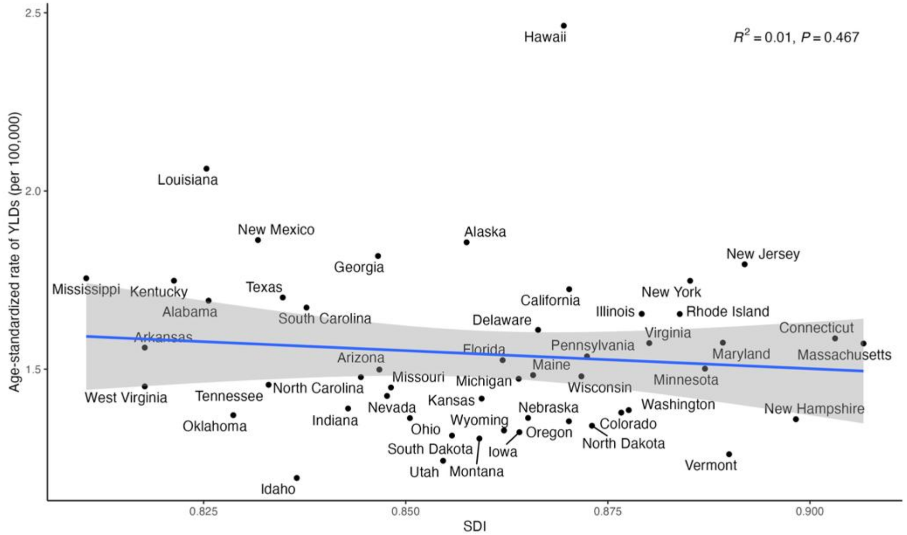

(E) YLLs

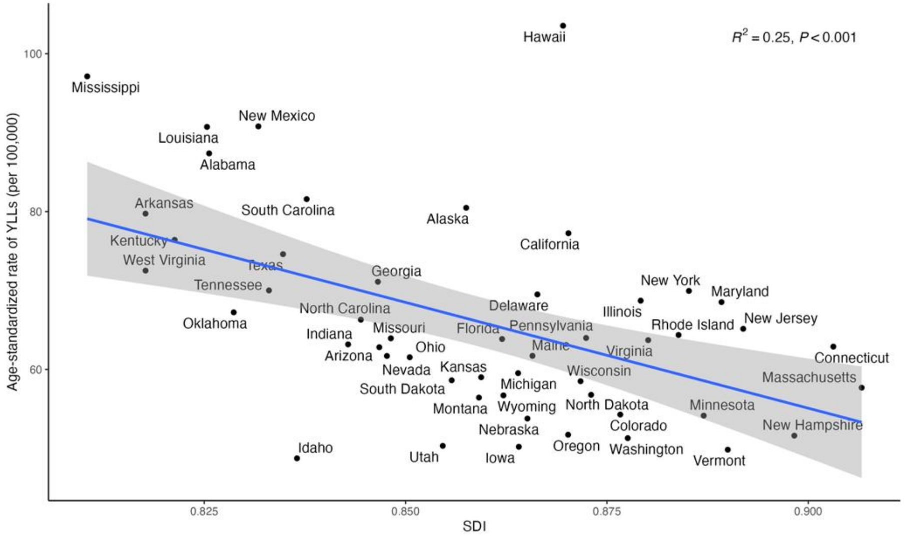

eFigure 6: Correlation between SDI and age-standardized rates of (A) incidence (B) death (C) prevalence (D) YLDs (E) YLLs for gastric cancer by state, 2021, United States
